# Extracellular matrix-associated gene expression in adult sensory neuron populations cultured on a laminin substrate

**DOI:** 10.1186/1471-2202-14-15

**Published:** 2013-01-30

**Authors:** Neva J Fudge, Karen M Mearow

**Affiliations:** 1 Division of BioMedical Sciences, Memorial University of Newfoundland, St. John’s, NL, Canada

**Keywords:** Peripheral nervous system, Dorsal root ganglion, Sensory neurons, Regeneration, Neurite growth, Extracellular matrix, Laminin, IB4, Microarray, Gene expression

## Abstract

**Background:**

In our previous investigations of the role of the extracellular matrix (ECM) in promoting neurite growth we have observed that a permissive laminin (LN) substrate stimulates differential growth responses in subpopulations of mature dorsal root ganglion (DRG) neurons. DRG neurons expressing Trk and p75 receptors grow neurites on a LN substrate in the absence of neurotrophins, while isolectin B4-binding neurons (IB4^+^) do not display significant growth under the same conditions. We set out to determine whether there was an expression signature of the LN-induced neurite growth phenotype. Using a lectin binding protocol IB4^+^ neurons were isolated from dissociated DRG neurons, creating two groups - IB4^+^ and IB4^-^. A small-scale microarray approach was employed to screen the expression of a panel of ECM-associated genes following dissociation (t=0) and after 24 hr culture on LN (t=24LN). This was followed by qRT-PCR and immunocytochemistry of selected genes.

**Results:**

The microarray screen showed that 36 of the 144 genes on the arrays were consistently expressed by the neurons. The array analyses showed that six genes had lower expression in the IB4+ neurons compared to the IB4- cells at t=0 (*CTSH, Icam1, Itgβ1, Lamb1, Plat, Spp1),* and one gene was expressed at higher levels in the IB4^+^ cells (*Plaur*). qRT-PCR was carried out as an independent assessment of the array results. There were discrepancies between the two methods, with qRT-PCR confirming the differences in *Lamb1, Plat* and *Plaur*, and showing decreased expression of *AdamTs1*, *FN*, and *Icam* in the IB4^+^ cells at t=0. After 24 hr culture on LN, there were no significant differences detected by qRT-PCR between the IB4^+^ and IB4^-^ cells. However, both groups showed upregulation of *Itgβ1* and *Plaur* after 24 hr on LN, the IB4^+^ group also had increased *Plat*, and the IB4^-^ cells showed decreased *Lamb1, Icam1* and *AdamTs1*. Further, the array screen also detected a number of genes (not subjected to qRT-PCR) expressed similarly by both populations in relatively high levels but not detectably influenced by time in culture (*Bsg, Cst3, Ctsb, Ctsd, Ctsl, Mmp14, Mmp19, Sparc*. We carried out immunohistochemistry to confirm expression of proteins encoded by a number of these genes.

**Conclusions:**

Our results show that 1B4^+^ and IB4^-^ neurons differ in the expression of several genes that are associated with responsiveness to the ECM prior to culturing (*AdamTs1, FN, Icam1, Lamb1, Plat, Plaur*). The data suggest that the genes expressed at higher levels in the IB4^-^ neurons could contribute to the initial growth response of these cells in a permissive environment and could also represent a common injury response that subsequently promotes axon regeneration. The differential expression of several extracellular matrix molecules (*FN, Lamb1, Icam*) may suggest that the IB4^-^ neurons are capable of maintaining /secreting their local extracellular environment which could aid in the regenerative process. Overall, these data provide new information on potential targets that could be manipulated to enhance axonal regeneration in the mature nervous system.

## Background

Dorsal root ganglia (DRGs) contain a heterogeneous population of neurons, which are often classified into three general groups on the basis of size, morphology, sensory modalities, trophic requirements and neurochemistry [1-4]. We have been investigating how the extracellular environment influences axonal growth using an *in vitro* system of mature DRG neurons and have found that not all populations of adult DRG neurons respond similarly to a permissive environment [5-7]. In our previous work we have shown that a population of small diameter nociceptive DRG sensory neurons (IB4^+^, characterized by their ability to bind *Griffonia simplificifolia* isolectin B4 (IB4)), do not show significant neurite growth on a LN substrate in the absence of added trophic factors [6], although they are capable of growth when GDNF is added in the presence of LN [6]. Others have also reported that IB4^+^ neurons have a decreased ability to regenerate *in vitro* compared to other DRG neuron populations, even after *in vivo* conditioning lesions that generally accelerate subsequent growth in culture [8,9]. Conditioning lesions also failed to stimulate regenerative central axon growth in IB4^+^ neurons *in vivo*[10]. The mechanisms for this difference are not fully known, but could involve selective expression of molecules involved in responsiveness to local environments in addition to known phenotypic characteristics [1,2,8,11,12].

The local nerve environment, particularly ECM components and receptors expressed by both neurons and Schwann cells, plays an important role in nerve regeneration [13-15]. Receptor such as integrins have been shown to be required for neurite outgrowth in adult sensory neurons *in vitro*[5,16-18], and we have previously shown that laminin-integrin interactions activate downstream signaling components in adult DRG neurons [19], although the resulting transcriptional events involved in neurite growth are not fully understood.

A number of studies have used large-scale whole genome microarray-based analysis to investigate the gene expression that is required for neurite outgrowth in regenerating adult DRG sensory neurons *in vivo*[20-23] and *in vitro*[24-26]. For example, Szpara *et al.*[26] reported that both superior cervical ganglia and DRG explants displayed a common upregulation of genes that encode ECM, basement membrane and cell adhesion proteins suggesting that remodeling of the local cellular environment is required for axon growth. However, total ganglia were used with no distinction between non-neuronal cells or neuron subtypes. Based upon the observed biological differences between IB4- and IB4^+^ neurons in our culture model, we sought to determine whether there was an ECM gene expression signature of LN-induced neurite growth. We employed a magnetic-bead based protocol to selectively isolate the IB4^+^ population from the total dissociated DRG neuron population (IB4^-^) [27]. Neurons that bind the IB4 lectin are generally thought to represent small nociceptive neurons which lack expression of the Trk and p75 neurotrophin receptors and are GDNF-responsive, while the IB4^-^ group include neurotrophin-responsive cells and have generally been characterized as Trk+, p75+, RT-97+ and CGRP+ neurons [1,2,7,28].

Small, membrane-based microarrays were employed to assess expression of ECM-associated genes in these neuronal populations. We hypothesized that there would be decreased expression of integrins and other ECM-related molecules in IB4^+^ neurons, thus potentially lowering the capacity of these neurons to respond to the permissive laminin environment.

Overall the data showed that 36 genes out of a possible 144 on the array were expressed by the DRG neurons, and of these, qRT-PCR quantitation showed 6 genes were differentially expressed in the IB4^+^ compared to the IB4^-^ neurons at the initial time point. In addition, the microarrays detected a number of genes that were relatively highly expressed in both populations of cells, although these were not further investigated by qRT-PCR. The results suggest that the genes expressed at higher levels in the IB4^-^ neurons may contribute to the initial growth response of these cells in a permissive environment and could also represent a common injury response that subsequently promotes axon regeneration. The differential expression of several extracellular matrix molecules may also suggest that the IB4^-^ neurons are capable of maintaining /secreting their local extracellular environment which could aid in the regenerative process. Our data support the supposition that ECM modeling through expression of proteolytic enzymes and matrix proteins is involved in axon outgrowth of adult neurons, and could be important in nerve regeneration *in vivo*.

## Results

### Neuronal growth response to a LN substrate

**Figure 1:**
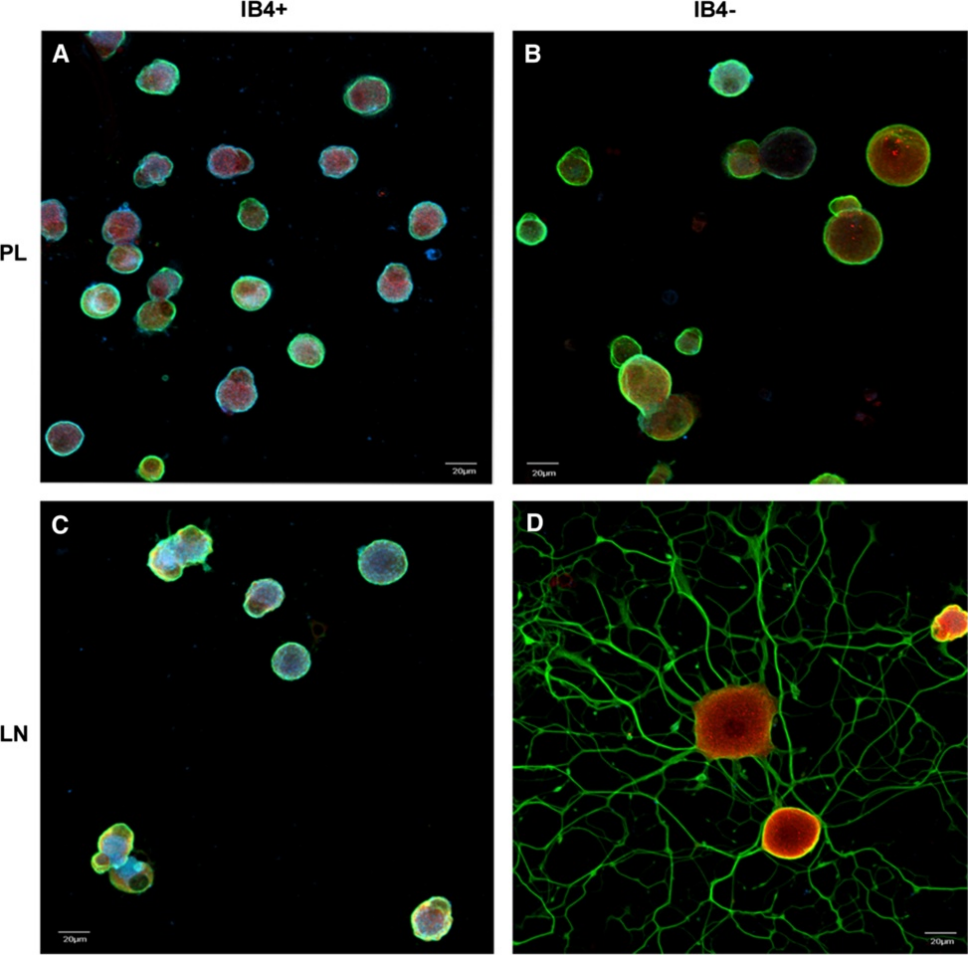
**IB4**^**+ **^**DRG neurons do not respond to a LN substrate with neurite growth.** DRG neurons were separated using isolectin B4 (IB4) coated magnetic beads (as described in the Methods) to produce two populations of neurons - IB4 selected (IB4^+^) and IB4^-^. Each cell group was plated on PL (**A**, **C**) and LN (**B**, **D**) coated culture dishes for 24 hr at 37°C. Cells were immunostained for βIII tubulin (green), integrin β1 (red) expression and IB4 lectin binding (blue) labeling. Note that while the larger IB4- neurons show extensive neurite growth, the IB4^+^ cells have no detectable growth. Scale bar-20 μm.

We have previously reported that IB4^+^ neurons have a reduced capacity to respond to a LN substrate [6]. To confirm this in our current studies, we collected DRGs from young adult rats, enzymatically dissociated them and cultured them on LN or poly-D-lysine coated (PL, control) culture dishes. The cells were fixed and immunostained for betaIII tubulin and labeled with biotinylated *Griffonia simplificifolia* isolectin B4 (IB4) followed by fluorescently tagged-streptavidin (Figure 1). After 24 hr culture on LN, the IB4^-^ DRG neurons exhibit neurite growth, with the large-diameter neurons having more elaborate neuritic networks than the small/medium neurons. In contrast, the IB4^+^ neurons did not show any significant growth (defined as neurites longer than one cell diameter). Neurites were not observed after 24 hr culture on PL in either population. Assessment of the number of neurite bearing- IB4^+^ cells was carried out and confirmed prior results (data not shown, see [5,7,27]).

### Gene expression analysis - microarrays

Neurons were dissociated and separated into IB4^+^ and IB4^-^ groups as described in the Methods [6,27]. Oligonucleotide filter-based microarrays were employed to investigate any differences in the expression of ECM associated genes between the selected IB4^+^ and the IB4^-^ populations either immediately after the selection procedure (t=0) or after a 24 hr culture on LN-coated culture dishes (t=24LN) (see Additional file 1: Table S1 for the complete gene list).

Of 144 ECM genes on the arrays, 36 genes were expressed by both neuron populations (Table 1; also Additional file 2: Figure S1; Additional file 3: Table S2). Eight of these genes (*Bsg, Cst3, Ctsb, Ctsd, Ctsl, Mmp14, Mmp19, Sparc*) encoding for lysosomal proteases, matrix metalloproteinases (MMPs) and regulators of MMPs were highly expressed in both populations at t=0 and t=24LN though not detectably different between the two populations (Additional file 3: Table S2, relative expression > 0.8 relative to highly expressed housekeeping genes (1.0), underlined). In addition, there were a number of other genes including different collagen isoforms, matrix and adhesion molecules, other proteolytic enzymes and an MHC molecule expressed by these neurons, although there were no significant differences between the IB4+vs the IB4- groups.

**Table 1:** 36 genes were expressed by both DRG neuron populations on microarrays


**Receptor/Adhesion molecules**	
***Icam1***	**Intercellular adhesion molecule**
*Itga5*	Integrin alpha 5
***Itgb1***	**Integrin beta 1**
*Itgb4*	Integrin beta 4
*Cdh1*	Cadherin 1
*Cdh2*	Cadherin 2
*Ctnnd1_predicted*	Catenin, delta 1 predicted
*Cntn1*	Contactin 1
*Cd44*	CD44 antigen
***Plaur***	**Plasminogen activator, urokinase receptor**
*Ncam1*	Neural cell adhesion molecule 1
*RT1-Aw2*	RT1 class Ib, locus Aw2
**ECM proteins**	
***Fn1***	**Fibronectin 1**
***Spp1***	**Secreted phosphoprotein 1 (osteonectin)**
***Lamb1_predicted***	**Laminin, beta 1 predicted**
*Col1a1*	Procollagen, type 1, α1
*Col4a1*	Procollagen, type 4, α1
*Col4a2_predicted*	Procollagen, type 4, α2 predicted
*Col5a3*	Procollagen, type 5, α3
*Col27a1*	Procollagen, type XXVII, α1
**Lysosomal proteases**	
*Ctsb*	Cathepsin B
***Ctsd***	Cathepsin D
***Ctsh***	**Cathepsin H**
*Ctsl*	Cathepsin L
**Matrix Metallopeptidases (MMPs)**	
*Mmp13*	Matrix Metallopeptidase 13
*Mmp14*	Matrix Metallopeptidase 14
*Mmp19*	Matrix Metallopeptidase 19
*Mmp24*	Matrix metallopeptidase 24
**Regulators of MMPs**	
*Bsg*	Basigin
*Cst3*	Cystatin 3
*Ctgf*	Connective tissue growth factor
*Sparc*	Secreted acidic cysteine rich glycoprotein
*Timp1*	Tissue inhibitor of metallopeptidase 1
**Other extracellular proteases**	
***Adamts1***	**A disintegrin-like and metallopeptidase (reprolysin type) with thrombospondin type 1 motif, 1**
*Hpse*	Heparanase
***Plat***	**Plasminogen activator, tissue**

9 of these (noted in *bold*) were differentially expressed between the IB4+ and IB4- neurons; these were further investigated using qRT-PCR. Underlined genes were expressed at relatively high levels (compared to array housekeeping genes) but did not differ in expression between the IB4+ vs the IB4- cells, and were therefore not subject to qRT-PCR analysis.

### Differences between IB4^+^ and IB4^-^ expression as assessed by microarray analyses

**Figure 2:**
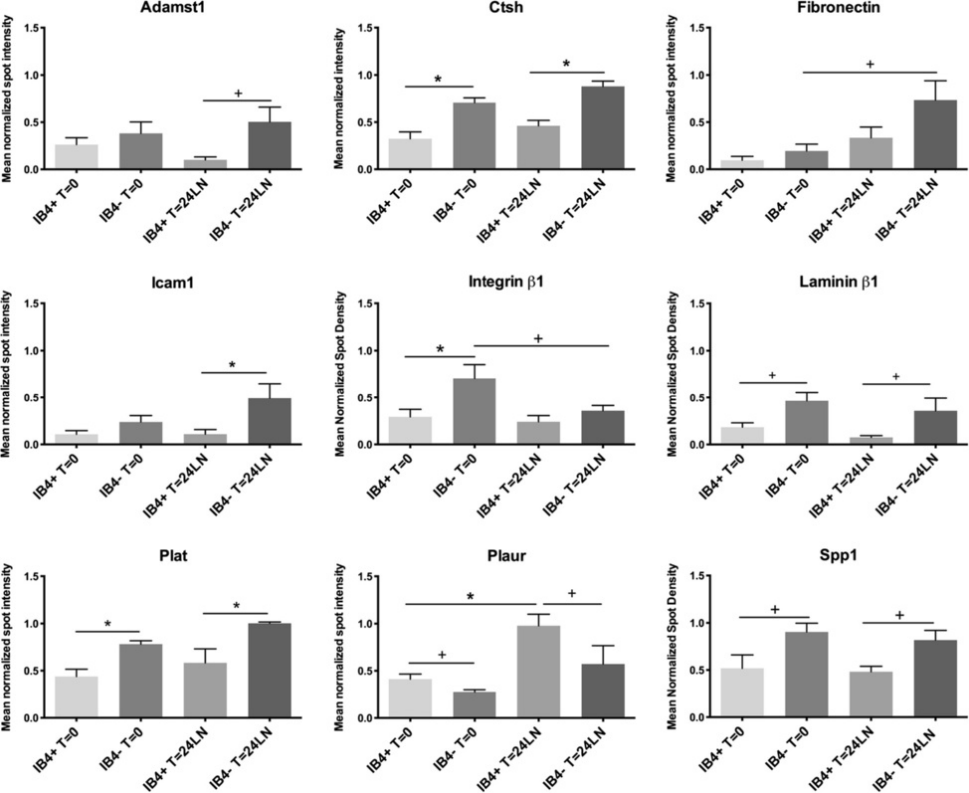
**Expression of selected genes detected as being differentially expressed between IB4**^**+ **^**and IB4**^**- **^**neurons by microarray analyses.** The graphs present the mean normalized spot density for each condition + SEM. Statistical significance was determined with ANOVA and Tukey post-hoc testing; * - p<0.05, + p<0.10.

Statistical analyses of the normalized data from the array screens showed that an additional set of genes were significantly different between the two populations at either t=0 or t=24LN with *CTSH, Itgβ1, Lamb1, Plat,* and *Spp1* being expressed at lower levels at t=0, and *Plaur* at higher levels in the IB4+ neurons; at t=24LN, *AdamTs1* and *Icam1* were significantly decreased, and *Plaur* was higher in the IB4+, while *Itgβ1* was no longer different. With respect to changes in each group with time in culture, *FN* was identified as an additional gene of interest. These data are summarized in Table 2 (see also Additional file 4: Table S3), and presented graphically in Figure 2. These genes were chosen for further assessment using qRT-PCR.

**Table 2:** **Differences in expression for IB4**^**+ **^**compared to IB4**^**- **^**neurons at t=0 and t=24LN as determined by each of the different assays employed**

**IB4**^**+**^	**T=0**			**T=24LN**		
**(compared to IB4**^**- **^**)**	**Arrays**	**qRTPCR**	**ICC**	**Arrays**	**qRTPCR**	**ICC**
AdamTs1	NS		NS		NS	NS
CTSH		NS	NS		NS	
Fibronectin	NS		NS	NS	NS	NS
Icam1	NS		--		NS	--
Integrin-β1		NS		NS	NS	
Laminin					NS	NS
Plat (tPA)			--		NS	--
Plaur					NS	
Spp1 (OPN)		NS			NS	

NS – not significantly different; *--* not determined. * - for ICC of Laminin analysis was of neurons cultured on PL substrate.

### Gene expression analysis - quantitative RT-PCR (qRT-PCR)

**Figure 3:**
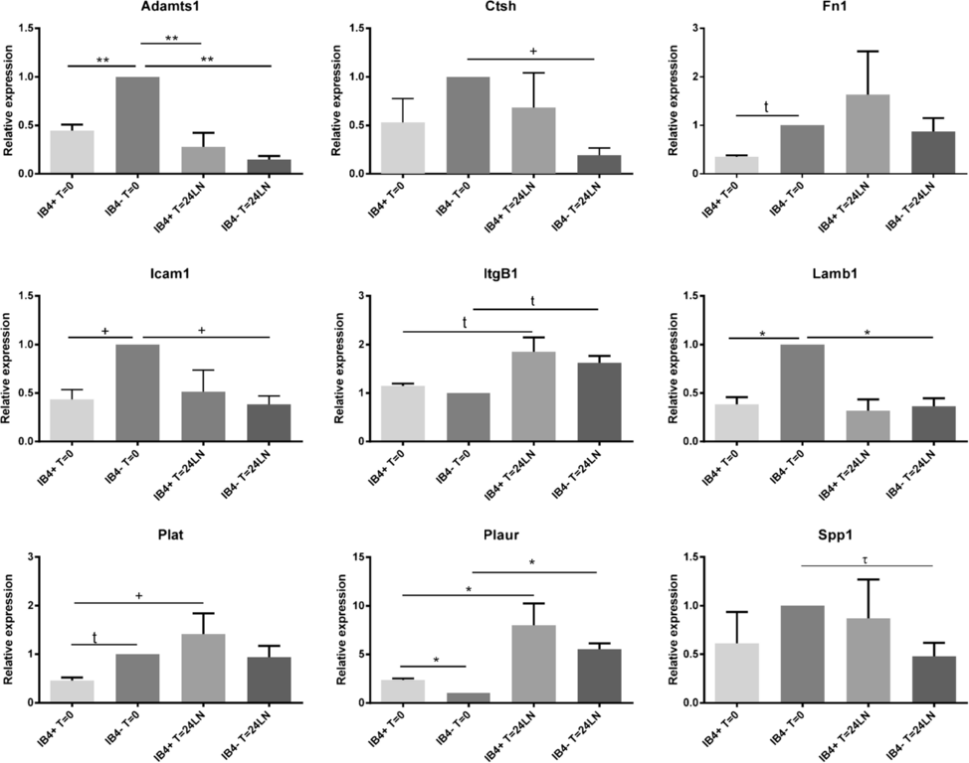
**qRT-PCR analysis of the 9 selected genes.** The graphs present gene expression relative (+SEM) to the IB4- t=0 condition (1); relative expression was calculated as outlined in the Methods. Statistical significance was determined with ANOVA and Tukey post-hoc testing; * - p<0.05, + p<0.10 using ANOVA; τ−p<0.05student t-test.

The nine genes noted above were subjected to further investigation using qRT-PCR. Pre-designed gene expression arrays (Taqman^R^ Assays-on-Demand™, see Methods for assay details) were employed to amplify the target genes and the data were expressed relative to IB4- at t=0 using a standard PCR protocol on a Fast Real-Time PCR platform. Relative quantity of gene expression was determined as outlined in the Methods section. The results are presented graphically in Figure 3 (see also Additional file 5: Table S4).

The differences between the groups at t=0 are presented in Table 2, where it can be seen that the PCR data did not fully agree with the array results. The IB4^+^ cells had lower levels of *AdamTs1, Fn1, Icam1, Lamb1, Plat* and higher expression of *Plaur* at t=0, but unlike the array data, there were no differences in *Itgb1, CTSH, Spp1* between the IB4^+^ vs IB4^-^ cells at this time point. At t=24 hr, there were no significant differences detected between the IB4^+^ vs the IB4^-^ groups (Table 2, Figure 3, Additional file 5: Table S4). With respect to differences in the IB4^+^ cells at t=0 vs t=24LN, *Plaur* was increased as were *Plat* and *Itgβ*1 (Table 3). In the IB4^-^ cells, *AdamTs1, Icam1* and *Lamb1* were decreased, while *Plaur* and *Itgβ1* were increased after 24 hr culture (Table 3).

**Table 3:** **Differences in expression for IB4**^**+ **^**compared to IB4**^**- **^**neurons at t=0 and t=24 as determined by each of the different assays employed**

	**IB4**^**+ **^**T=24 vs T=0**			**IB4**^**- **^**T=24 vs T=0**		
	**Arrays**	**qRTPCR**	**ICC**	**Arrays**	**qRTPCR**	**ICC**
AdamTs1	NS	NS		NS		
CTSH	NS	NS		NS	NS	
Fibronectin	NS	NS			NS	
Icam1	NS	NS	--	NS		--
Integrin-β1	NS		NS			NS
Laminin	NS	NS		NS		
Plat (tPA)	NS		--	NS	NS	--
Plaur			NS	NS		NS
Spp1 (OPN)	NS	NS	NS	NS		

NS – not significantly different; *-* not determined. * - for ICC of Laminin, analysis was of neurons cultured on PL substrate.

Of note is the fact that the qRT-PCR data did not completely agree with the array results in terms of the distinction between the IB4^-^ and IB4^+^ cells. This particularly evident at t=24LN where there were no significant differences between the populations, with relative expression levels being similar (Figure 3). The TaqMan^R^ assays also picked up differences within each group over time that that did not correspond with, nor were detectable in, the array assays. The IB4^+^ group had increased expression of *Itgb1*, *Plat* and *Plaur* after 24hr culture, the IB4^-^ group also had increased *Itgβ1* and *Plaur*, but decreased expression of *AdamTs1*, *Icam1* and *Lamb1*. The lack of significant difference between the populations at 24 hr could be a result of expression changing between t=0 and t=24 in the individual groups, such that increased expression in the IB4^+^ cells from t=0 to t=24 and the decreases in the IB4^-^ group over the same period could offset differences between the populations (Figure 3). Further discussion of the discrepancies between the 2 assay types is found in the Discussion.

### Cellular protein expression

**Figure 4:**
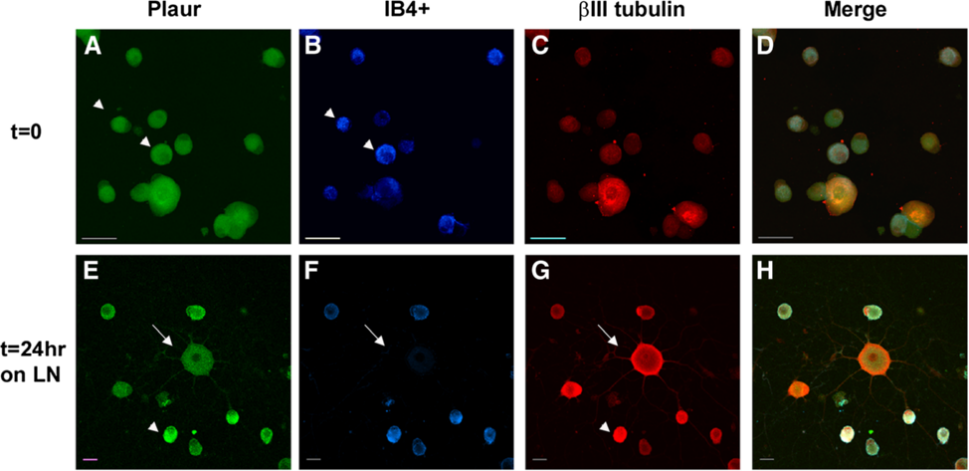
**Plaur protein expression in DRG neurons.** Dissociated DRG neurons were immunostained for Plaur (green), neuron-specific βIII tubulin (red) and IB4 lectin labeling (blue) at t=0 (**A-D**) and t=24LN (**E-H**). While both IB4^+^ and IB4^-^ neurons express Plaur, densitometric analyses (see Supp. Fig 2) showed that the expression was higher in the IB4^+^ cells. Scale bar-A-D, 50 μm; E-F, 20 μm.

**Figure 5:**
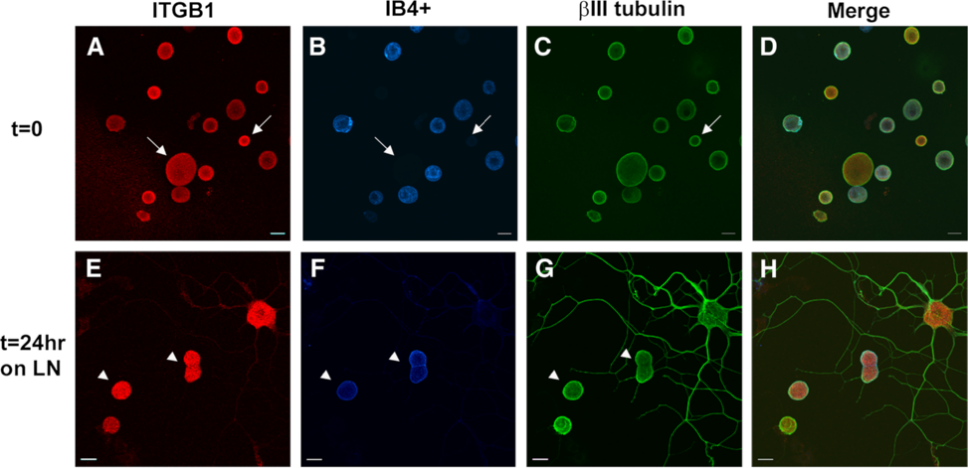
**Integrin β1 protein expression in DRG neurons.** Dissociated DRG neurons were immunostained for integrin β1 (red), neuron-specific βIII tubulin (green) and IB4 lectin labeling (blue) at t=0 (**A-D**) and t=24LN (**E-H**). Both IB4^+^ and IB4^-^ neurons express Integrin β1 and densitometry indicated that the IB4^+^ cells had somewhat lower levels of expression. Scale bar-20 μm.

**Figure 6:**
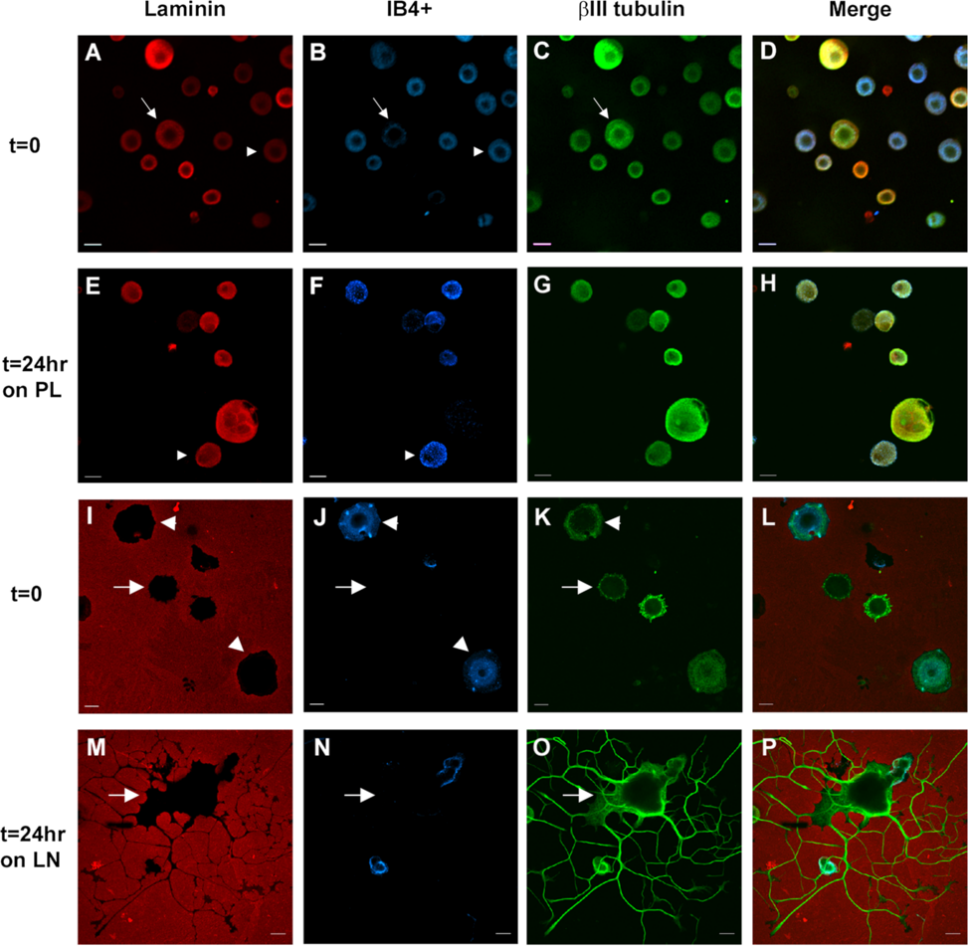
**DRG neurons express LN.** Dissociated DRG neurons were immunostained for laminin (red), neuron-specific βIII tubulin (green) and IB4 lectin labeling (blue) at t=0 (**A-D**) and t=24 hr after plating on poly-lysine (**E-H**). Neurons cultured on poly-lysine were used to detect the LN expression, as it was not possible to detect the cellular LN signal above the stained LN substrate. ICC quantitation showed that IB4^+^ cells had lower levels of LN expression at t=0 compared to the IB4^-^ cells. Scale bar-20 μm.

**Figure 7:**
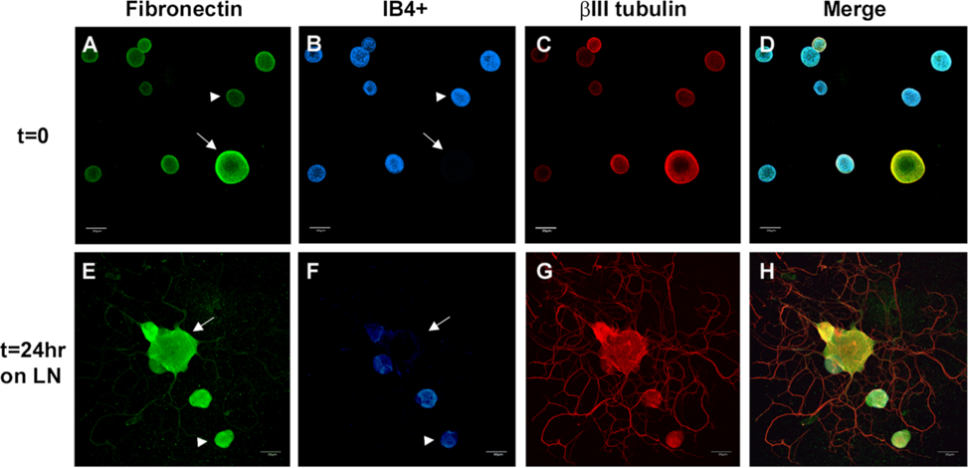
**FN protein expression in DRG neurons.** Dissociated DRG neurons were immunostained for fibronectin (green), neuron-specific βIII tubulin (red) and IB4 lectin labeling (blue) at t=0 (**A-D**) and t=24 hr after plating on LN (**E-H**). Both populations of cells express FN with no obvious difference detected by densitometry at t=0, although after 24 hr on LN, an increase in expression was observed for both populations. Scale bar-30 μm.

**Figure 8:**
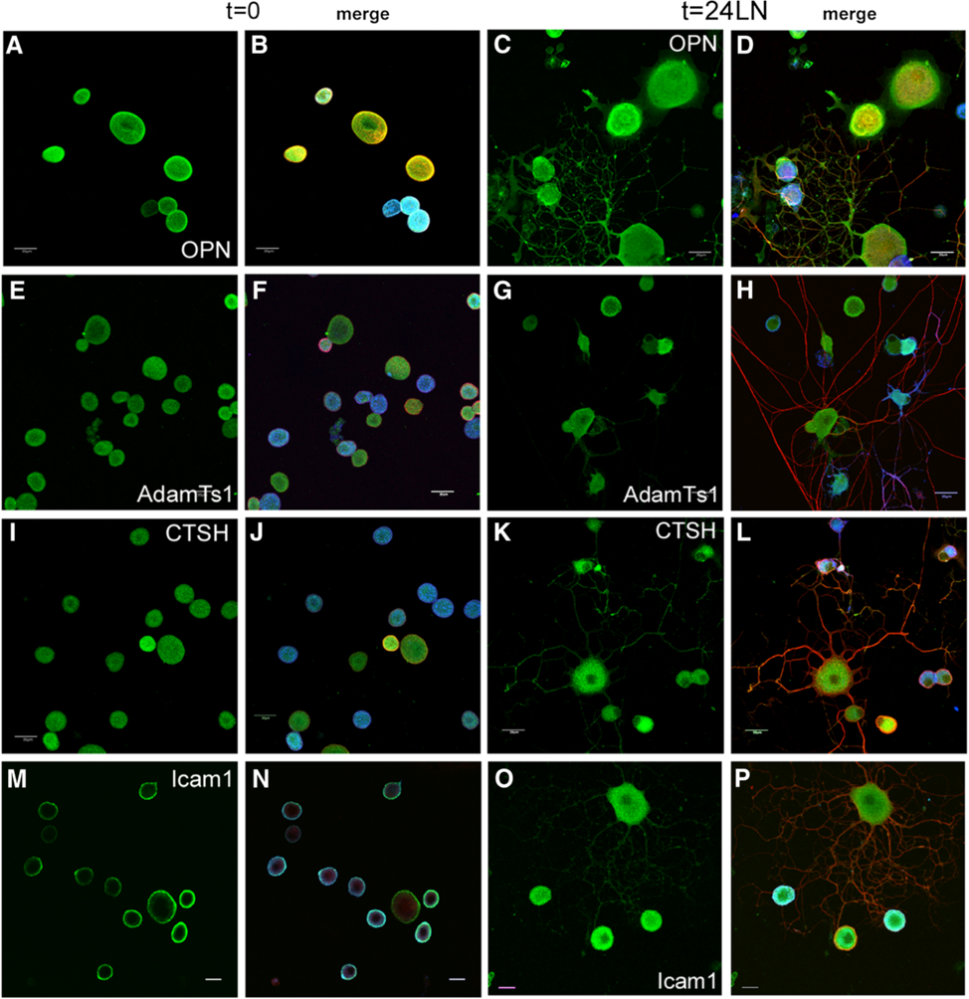
**Expression of OPN, CTSH, AdamTs1 and Icam.** ICC was used to detect the expression of osteopontin (**A**, **C**, green), AdamTs1 (**E**, **G**, green), CTSH (**I**, **L**, green), Icam (**M**, **O**, green); merged images show βIII tubulin (red, **B**, **D**, **F**, **H**, **J**, **L**, **N**, **P**) and IB4 binding neurons (blue) at t=0 and t=24LN. Densitometric analyses are presented in Supp Fig 2. Scale bar-A-L, 30 μm; M-P, 20 μm.

Fluorescent immunocytochemistry (ICC) and confocal microscopy were utilized to investigate the cellular expression in DRG neurons. Total dissociated DRG neuron populations (rather than undertaking the selection protocol) were plated on chambered glass slides coated with either PL or LN, and incubated for 45 min to allow cell adherence (nominally referred to as t=0) or for 24 hr (t=24 LN). All images were acquired with the same imaging parameters. In order to distinguish the 2 main populations, the immunostained neurons were additionally labeled with the IB4 lectin. Analysis of immunostained neurons was carried out using confocal images converted to grey scale and subjected to densitometry using Image J to provide some estimation of the differences between populations at each time point (see Additional file 6: Figure S2). Figures 4, 5, 6, 7, 8 present representative images of Plaur (Figure 4), Integrin β1 (Figure 5), LN (Figure 6), FN (Figure 7) and OPN, AdamTs1, CTSH, and Icam (Figure 8). Both the IB4^+^ and IB4^-^ displayed detectable levels of all proteins tested; densitometry results are summarized in Table 2–3 and displayed graphically in Additional file 6: Figure S2.

Plaur (Figure 4) was present in both IB4^+^ and IB4^-^ neurons, with higher levels detected in the IB4^+^ cells. Integrin β1 (Figure 5) was also expressed in both groups, with the IB4^+^ cells showing decreased expression. LN (Figure 6) was also expressed by both groups, with lower expression in the IB4^+^ cells. Note that for LN immunostaining we assessed cells plated on PL (Figure 6A-H), as the background staining of the LN substrate with the LN antibody made it difficult to visualize neuronal expression. Imaging of LN immunostaining on the LN substrate resulted in ‘negative-like’ patterns of cell attachment and neurite growth (Figure 6I-P), likely due to accessibility of the antibody to the substrate underlying the neurons, although it might also indicate some proteolysis of the LN substrate. There were no differences in FN expression (Figure 7) between IB4^+^ and IB4^-^ neurons at t=0 but expression was increased in both groups at t=24LN. Both groups of neurons expressed AdamTs1, CTSH, OPN and Icam (Figure 8), with no differences between the groups at t=0, except for OPN which was higher in the IB4^+^ cells. At 24 hrs, AdamTs1 increased in both groups, OPN increased in the IB4^-^ cells and CTSH decreased in the IB4^+^ group (Table 3, Additional file 6: Figure S2).

While the densitometry analyses indicated differences in staining intensity, this assessment is at best only an estimation of protein expression, and was intended to ensure that the neurons did express the proteins. The differences detected did not agree with the quantitation of gene expression differences.

### Immunohistochemistry of DRG sections

**Figure 9:**
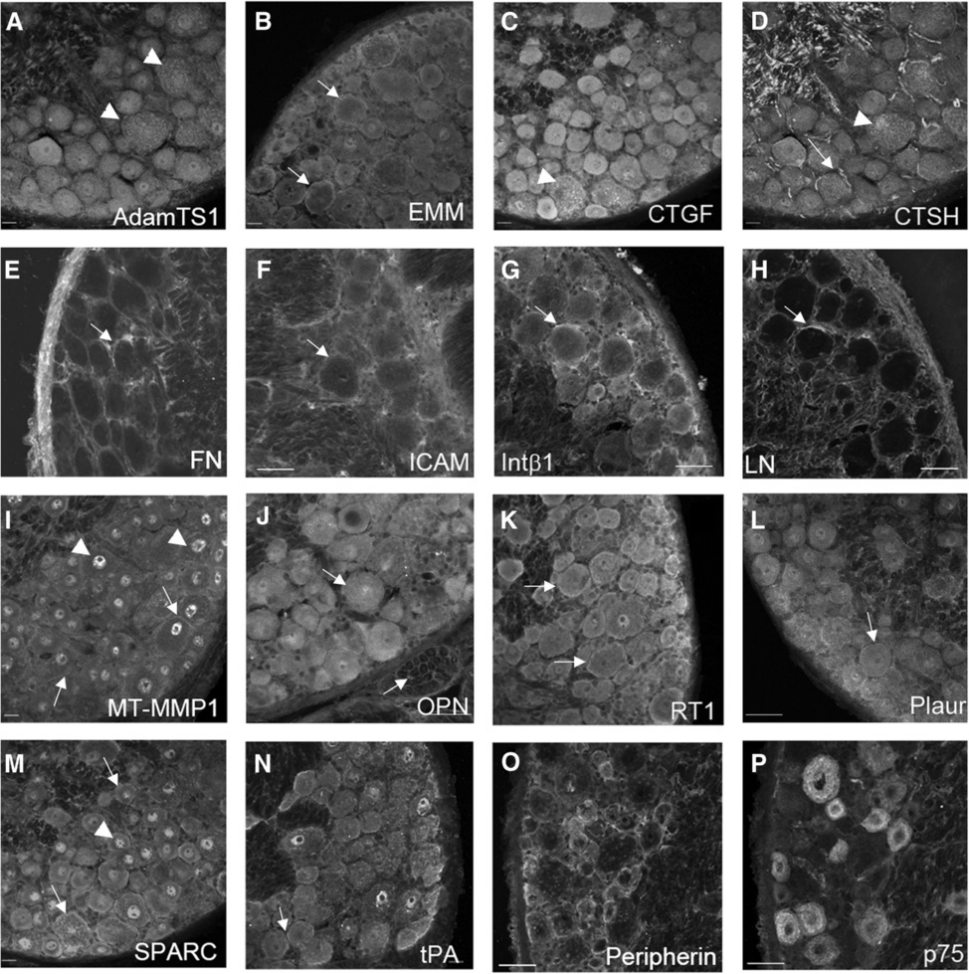
**Immunohistochemisty of selected proteins in adult rat DRG cryosections**. IHC and confocal microscopy was used to investigate expression of proteins encoded by a number of genes noted to be expressed at robust levels in the dissociated neurons, including those that have not been previously described in DRG neurons. Panels **A** – AdamTs1; **B** - Emmprin (Basigin); **C** – CTGF; **D** – CTSH; **E** - Fibronectin; **F** – Icam; **G** – Integrin β1; **H** – Laminin; **I** – MT-MMP1 (MMP19); **J** – Osteopontin (Spp1); **K** – RT-1 (RT-AWs); **L** – Plaur; **M** – SPARC; **N** – tPA (Plat); **O** – Peripherin; **P** – p75 neurotrophin receptor. Scale bar – 50 μm.

We also undertook to examine proteins encoded by some of the genes detected by the arrays (including those not previously described in DRG neurons) by IHC of DRG cryosections (Figure 9). FN, ICAM, Integrinβ1 and LN appeared to be associated with the surface of the neurons as well as in the surrounding non-neuronal cells (Figure 9E-H, arrows). AdamTs1, CTGF and CTSH (Figure 9A, C, D) were expressed primarily in the cytoplasm and appeared granular in nature (arrowheads), while CTSH also labeled axons within the neuropil (Figure 9D, arrow). Basigin (also known as Emmprin), which is a membrane associated protein, appeared to be enriched on the cell surface, but also present in the cytoplasm (Figure 9B, arrow). Osteopontin, Plaur and Plat appeared to be expressed in the neurons, as well as being associated with non-neuronal cells (Figure 9J, L, N, arrows). Interestingly in addition to cell surface labeling, both MT-MMP1 (MMP14) and SPARC also displayed nuclear labeling (Figure 9I, M), which did not appear to be artefactual, as it was present with several fixation and permeabilizing protocols. The MT-MMP1 nuclear staining in particular was punctate and quite distinct (Figure 9I, arrowheads), but the role of MT-MMP1 in the nucleus is not clear. RT1 (which was relatively highly expressed in both populations at t=24LN) detects a non-classical MHC-class 1 antigen specific to rat cells [29] and the staining shows enrichment on the surface of the neurons (Figure 9K, arrows). Supplemental Figure 3 and 4 present IHC for these same proteins, but showing double staining for pairs of antibodies along with IB4-labelling of the sections.

## Discussion

### ECM-related gene expression by DRG neurons

We hypothesized that there would be gene expression differences between the IB4^+^ and IB4^-^ cells given the observed growth responses in culture. Our results support the hypothesis to some extent, noting that there were intrinsic differences (eg., at t=0) in gene expression as assessed by qRT-PCR, with the IB4^+^ cells showing lower expression of *AdamTs1, Fn, Icam1, Lamb1, Plat,* but higher expression of *Plaur*. While several of these are clearly involved in axonal growth or regeneration in the peripheral nervous system (*Fn1* (fibronectin) [30-34], *Icam1*[35], *Lamb1*[18], *Plaur* and *Plat*[13,36,37]), their expression in neurons has been less well described.

### Discrepancies between the microarray and qRT-PCR results

As noted in the Results, the qRT-PCR data did not completely agree with the array results in terms of differences between the IB4^-^ and IB4^+^ cells. This was particularly evident at t=24LN where there were no significant differences between the populations (Figure 3). The TaqMan^R^ assays also picked up differences within each group over time that that did not correspond with, nor were detectable in the array assays. There are a number of potential explanations for the discrepancies. A basic issue relates to the assay platforms and the sequences probed by the different methods. Specific primer sequences are not available for the TaqMan assays nor does the microarray platform (SABiosciences) provide sequence information with respect to the panels of oligonucleotides used to create the arrays, thus caution is required in interpreting or comparing results from these two very different approaches. Quantitative RT-PCR assays are more reliable and sensitive to changes in mRNA expression although such assays can also be subject to caution (http://miqe.gene-quantification.info). It is also possible that the discrepancies could be due to the different platforms probing different regions of the genes or that distinct isoforms are being expressed by the populations. For example, Spp1/OPN exists as three isoforms derived from alternative splicing and these have been reported to have differing effects on cellular behaviour including regulating expression of Plau, the ligand for Plaur [38]. Several splice variants of Plaur/uPAR are also reported in cancer with differing expression and biological activity in malignant vs non-malignant cells [39].

The differences in protein expression detected by ICC did not agree with the quantitation of gene expression differences, although one would not necessarily expect a direct correlation [40,41]. Post-transcriptional mechanisms including translation and post-translational modification as well protein turnover will modulate protein steady state levels [41]. Post-transcriptional mechanisms have been suggested to contribute to discrepancies in plasminogen enzyme activities compared to mRNA levels in neonatal DRG neurons [42]. The difference could also point to post-translational modifications or the presence of proteins translated from splice variants, since alternative-spliced isoforms have been described for several of these targets as noted above, albeit in cancer or metastasizing cells (eg., [39,43]). In any case, the ICC provided confirmation of the genes, including those not previously described or detected at the protein level in the DRG (eg., Bsg, AdamTs1 [44]; MMP 14, MMP19, RT-Aw2, Ctgf).

Technical and biological variability between the different assays contribute to the observed discrepancies. Thus, while the microarray approach was useful for screening purposes, the quantitative changes in gene expression between the populations that we have presented are based upon the data obtained from qRT-PCR analyses.

### The tissue plasminogen (uPAR/Plaur and tPA/Plat) system in sensory neurons

Many cells types use extracellular proteolysis to modify the ECM or cell-cell contacts in order to respond to environmental cues. Enzymes involved include MMPs and members of the plasminogen activator/plasmin system. Two types of plasminogen activators are tPA/Plat and uPA/Plau or urokinase; uPAR/Plaur is the receptor for uPA. Both PAs convert plasminogen into active plasmin, which is a protease with targets that include ECM components, growth factors and cellular receptors [45].

Our data show that *Plat* was expressed at higher levels in the IB4^-^ cells at t=0, but was increased in the IB4^+^ cells by 24 hr. tPA plays a key role in growth and regeneration in the nervous system [45], with tPA enhancing sensory axon regrowth in the spinal cord in a model of spinal cord injury [46]. LN has been shown to be degraded by tPA activation of plasminogen and to be necessary for PC12 cell neuritogenesis on LN [47]. Plat may play a similar role here in the LN-induced growth of the IB4^-^ neurons.

Our results showed that *Plaur* was expressed at higher levels in the IB4^+^ neurons compared to the IB4^-^ cells at t=0; both were increased after 24 hr. The relative increases in *Plaur* with time in culture were several-fold higher than any of the other genes investigated by qRT-PCR; similar large increases have been observed in cultures of neonatal DRG neurons [42]. The reason for this is unclear, particularly with the suggested role of *Plaur* in nerve regeneration, and the reported lack of growth of the IB4^+^ cells *in vitro* and *in vivo*[5,8,9]. However, considering that these cells are able to undergo neuritogenesis in culture when GDNF is added, it suggests that they do have the capacity to regenerate, provided the appropriate growth factors are also made available.

The *Plaur* gene encodes for a cell surface receptor (Plaur in rats, uPAR in mice and humans) that binds urokinase plasminogen activator (uPA), resulting in the conversion of plasminogen to plasmin to promote pericellular matrix degradation [48]. In cancer, the plasminogen activator system has been extensively studied for its role in cell migration and invasion [49]. Interestingly, uPAR has been reported to be necessary for nerve growth factor (NGF)-induced neurite extension in PC12 cells [50]. Furthermore, an increase in *Plaur/uPAR* expression in sensory neurons after injury has been previously described. Hayden and Seeds [42] showed that *uPAR* and *uPA* mRNA levels significantly increased in neonatal mouse DRG neurons during the first day of culture. In subsequent *in vivo* nerve injury experiments, Siconolfi and Seeds [36] reported that DRG neuron *uPAR* mRNA expression increased by 8 hr post-injury, while uPA did not increase until day three. These studies suggested that the plasminogen activator system was likely to play a key role in peripheral nerve regeneration. The role of uPAR in peripheral nerve function and regeneration has only recently been more completely elucidated. In a recent report, using uPAR null mice, Previtali and colleagues have shown that lack of uPAR inhibits nerve regeneration following injury, which was attributed to a decrease in fibrinolytic activity in the damaged nerve and concomitant deposition of fibrin and vitronectin [13]. This work suggests that uPAR/Plaur is key to the appropriate remodeling of the ECM necessary for nerve regeneration [13], although no differences in the neuronal subtypes were investigated.

In addition, plasminogen-independent roles for *Plaur* have been explored. For example, Plaur has also been shown to interact with cell surface molecules such as integrins [51-55] and mediate MEK/ERK signaling through FAK or Src [56-58]. Plaur also regulates the activity of MMPs after sciatic nerve crush [59]. Thus, the increase in *Plaur* mRNA and protein in the absence of uPA expression may point to a uPA-independent role(s) in these cultured DRG neurons. Our data, combined with the previous literature, suggest that *Plaur* expression may be involved in essential interactions with the ECM in these neurons. However, further experiments are necessary to investigate whether the increased expression of *Plaur* in the IB4^+^ cells plays any part in the reported diminished ability of these neurons to regenerate compared to other classes of DRG neurons [2,6,8-10].

### Integrin expression in DRG neurons

The microarrays detected that integrins β1, β4 and α5 were consistently expressed by these neurons. We have previously detected expression of α1, α5, and α3 integrins along with β1 by ICC, and have shown that β1 is required for LN-induced neurite growth [5]. While Integrin β1 was found to be decreased in the IB4^+^ cells by both array and ICC analysis, the qRT-PCR did not detect significant differences at t=0 between the 2 groups. However, after 24 hr culture on LN, increased gene expression in both the IB4^+^ and IB4^-^ cells was detected by qRT-PCR. While our initial hypothesis was that differences in integrin expression might contribute to the relatively rapid initial growth from IB4^-^ cells compared to IB4^+^, these data indicate that this is not the only factor in the growth response.

Other studies have pointed to an important contribution of integrin α7β1 expression to the regeneration promoting effects of preconditioning lesions [60,61]. However, α7 is not expressed by IB4^+^ neurons, which have a reduced regenerative capacity, nor does forced expression of α7 lead to any rescue of this lack of growth [8,9,17]. Integrin α7 was not detected by the arrays, although standard RT-PCR showed that the mRNA was expressed in both populations (data not shown). However, as we have previously been unable to detect α7 by ICC in our cultures using several different antibodies, it is possible that while the mRNA is present, there is a low level of translation not detectable by our methods.

### Other genes of interest detected on the microarray screen

Our initial approach was to use the arrays as a platform to screen for genes that were differentially expressed between two populations of DRG neurons and might conceivably contribute to the differences in neurite growth induced by LN. This approach identified the genes that were then further assessed by qRT-PCR as noted above. A number of other genes were identified that displayed relatively high levels of expression (compared to the housekeeping genes) in both sets of neurons, but were not detectably changed over time in culture. Several of these (*Bsg*, *Sparc*, *Adamts1*, *Mmp14*) have only recently been described in embryonic DRG neurons using a similar microarray approach, but neither Bsg nor AdamTs1 was detected at the protein level [44]. For others (*Mmp19*, *Ctgf*, *RT1-Aw2*), to our knowledge it is the first time that they have been reported to be expressed in adult DRG neurons, although *Ctgf* was picked up in a large scale screening of genes associated with outgrowth in embryonic DRG, using RNA isolated from whole ganglia [26]. IHC to detect a number of these proteins showed that they are expressed in the ganglia both in neurons and in associated non-neuronal cells DRGs (Figure 9; also Additional files 7-8: Figures S3-4).

Several of these genes have been studied for their role in ECM remodeling in other cell types. Remodeling of ECM components clears a path for cell migration and plays a role in peripheral nerve regeneration [13,62]. Of interest, MMP14 (also known as MT1-MMP) is a membrane-bound MMP that regulates the turnover and integrin-mediated endocytosis of fibronectin [63], and our results show neuronal and nuclear expression in the DRG sections. BSG (also known as EMMPRIN) is a type 1 integral membrane receptor that has the ability to complex with integrin α3β1 [64] and induce MMP expression [65,66]. It has also been shown to mediate neuron-glial interactions important in the development of the Drosophila eye [67]. IHC results demonstrate expression in DRG neurons, with an apparent cytoplasmic and cell surface localization. As these genes were robustly expressed in DRG neurons, they could be important of the response of DRG neurons to their extracellular environment *in vivo*.

Another gene not previously reported as being expressed in DRG neurons is *RT1-Aw2*. *RT1-Aw2* is an MHC class I molecule [29], and MHC class I molecules have recently been implicated in the immunoregulatory functions of synaptic plasticity after nerve injury [68] and in neuroinflammation [69]. This could suggest that *RT1-Aw2* plays a key role in antigen presentation and neuro-immune interactions in these cultured adult neurons. RT1 is expressed in DRG neurons and appears to be enriched on the cell surface (Figure 9K).

The genes (*Bsg, Cst3, Ctsb, Ctsd, Ctsl, Mmp14, Mnp19, Sparc*) detected by the microarray screen as being relatively highly expressed are involved in the degradation of extracellular and intercellular proteins and could play a role in the response of the neurons to ECM cues in axonal growth [70]. Because there were no differences observed between the populations on the arrays, these genes were not subjected to further analyses by qRT-PCR. However, given the discrepancies observed between the two assays, we will investigate this further. Future studies will focus on these genes to understand what role they play in ECM remodeling that could promote axonal growth in *in vitro* and *in vivo* systems.

In summary we have investigated whether the observed growth differences between IB4+ and IB4- neurons were associated with any differences in the expression of genes associated with the ECM. Several differences were observed, which may play a role in the growth response or represent a general response to cellular injury.

## Conclusions

Our results show that 1B4^+^ and IB4^-^ neurons differ in the expression of several genes that are associated with responsiveness to the ECM prior to culturing (*AdamTs1, FN, Icam1, Lamb1, Plat, Plaur*). The data suggest that genes expressed at higher levels in the IB4^-^ neurons could contribute to the initial growth response of these cells in a permissive environment and could also represent a common injury response that subsequently promotes axon regeneration. The differential expression of several extracellular matrix molecules (*FN, Lamb1, Icam*) suggests that the IB4^-^ neurons are capable of maintaining/secreting their local extracellular environment which could aid in the regenerative process. Overall, these data provide new information on potential targets that could be manipulated to enhance axonal regeneration in the mature nervous system.

## Methods

### DRG neuron separation and culture

Animal procedures were approved by the Animal Care Committee at Memorial University of Newfoundland in accordance with The Canadian Council on Animal Care (CCAC). For each biological replicate, DRGs were collected from six young adult Sprague Dawley rats (4–7 weeks old), cleaned of attached nerve roots and connective tissue, pooled and enzymatically dissociated using our standard protocol [27]. Approximately 95% of non-neuronal cells were removed using a 4 × 4 minute centrifugations at 50 x g in a 15 ml centrifuge tube prior to the separation procedure. IB4^+^ binding DRG neurons were separated from the total DRG neuronal population using our established magnetic separation protocol that utilizes Streptavidin-Dynabeads coated with biotinylated isolectin B4 from *Banderaea simplicifolia* (Sigma, cat#L2140) to produce two populations of DRG neurons [27]. Briefly, neurons are incubated with the lectin-coated beads, and neurons that bind the lectin are subsequently collected using a magnet (IB4^+^ neurons). The IB4^+^ selected cells are detached from the beads after a DNase treatment which degrades the DNA linker that attaches the streptavidin to the beads. The supernatant contains the remaining neuronal populations, the IB4^-^ group. Our optimized protocol results in neuronal enrichment with less than 1-5% non-neuronal cells. We visually assessed the composition of the IB4^+^ and IB4^-^ cell groups to ensure that this was the case and discarded any samples that were considered above this limit. The usual time from euthanasia to cell plating was on the order of 2–2.5 hrs. We have noted that binding of the lectin to the neurons as would occur in the separation protocol does not appear to account for any differences in growth, as the lack of growth of the IB4+ cells is detectable with or without the selection procedure. We and others have shown that IB4+ cells have a reduced ability to regenerate both *in vivo* and *in vitro* in studies where lectin-binding is employed to identify the cells after fixation, and in the absence of any separation protocol [6,8,9].

For mRNA analysis, t=0 samples were collected from IB4^+^ and IB4^-^ populations of neurons immediately after the separation procedure for total RNA extraction. Alternatively, t=24LN samples were collected from the two populations after 24 hr culture on laminin (LN, 20 μg/ml) coated 35 mm culture dishes at 37°C. Neurons were maintained in serum-free Neural Basal Media (NBM) with B27 supplement and mitotic inhibitors. For immunocytochemical analysis of cellular protein, total (not separated) DRG neuronal populations were cultured in NBM either on poly-D-lysine (PL) coated 35 mm culture dishes for 45 min (t=0), or on LN coated dishes for 24 hr (t=24LN).

### RNA collection and preparation

Total RNA was extracted from each sample using an RNeasy plus micro kit (Qiagen). For t=0 samples, the IB4^+^ and non-selected neurons were pelleted after the separation procedure for 5 minutes at 300 x g. The supernatant was then removed and the pellet was resuspended in 350 μl of Buffer RLT. For t=24LN samples, the culture medium was removed and 350 μl of Buffer RLT was added to each well of a 6-well culture plate to directly lyse the neurons. The well was then scraped with a rubber policeman and the sample was transferred from the well to a 1.5 ml centrifuge tube. The lysed samples were processed through the remainder of the RNeasy Plus Micro protocol as per the manufacturers manual. Total RNA was quantified using a NanoDrop 1000 spectrophotometer (ThermoScientific) and stored at −80°C.

### cRNA synthesis and labelling

Biotinylated cRNA was prepared using the True-Labeling-AMP 2.0 kit (SABiosciences) using 600 ng of total RNA. Labeled cRNA was purified employing the ArrayGrade cRNA Cleanup kit (SABiosciences) and quantified with a NanoDrop 1000 spectrophotometer (ThermoScientific).

### GEArray hybridization

Gene expression was analyzed using Gearray oligo microarrays (SABiosciences) that were designed to analyze the mRNA expression levels of genes that encode for extracellular matrix attachment and adhesion molecules and remodeling enzymes (extracellular and lysosomal proteases) that were specific for rat tissues (Additional file 1: Table S1). These arrays are nylon membranes containing a printed matrix of 60-mer oligonucleotide probes specific to each gene. During the course of the experiment, the manufacturer created an updated version of the array. Thus two arrays were used for the experiments (ORN 0.13 and ORN 0.13.2). Although the arrays individually analysed the expression of 133 genes, due to small differences between the gene lists of each array, we ultimately analysed 144 genes. Three experiments were performed with ORN 0.13 and four experiments with ORN 0.13.2. This caused the number of biological replicates to vary for each gene. GEarray membranes were hybridized with the biotin-labeled cRNA target and 0.75 ml of GEarray hybridization solution overnight at 60°C. The hybridization solution was then removed and the array membrane was washed with 2XSSC, 1% SDS for 15 min at 60°C. Blocking and detection were performed at room temperature using the Chemiluminescent Detection kit (SA Biosciences). The membrane was exposed to film for 20 sec to 5 min, and films were developed and scanned to create digital images. Gene expression was indicated by the density of a hybridized “spot” for each gene. The digital images for each array were uploaded to the SABiosciences online GEarray Expression Analysis Suite.

This software calculated the intensity of the hybridization spots for each gene on the array. Spot intensities (densitometry) for each gene were subtracted from background and normalized to housekeeping gene expression levels (1.0) to calculate the absolute value of expression for each gene. Gene expression levels ranged from 0.02 (faint spot, very low expression) to 1.34 (bleeding spot, very high expression). The housekeeping genes [peptidylpropyl isomerase A, ribosomal protein L32, lactate dehydrogenase, aldolase A, glyceraldehydes-3-phosphate dehydrogenase and a biotinylated artificial sequence 2 complementary sequence] were located on the top left corner and along the lower edge of the array. The absence of the expression of particular genes may indicate that the expression level is below the detection limit of the array technique. Relative quantities were determined by expressing normalized values relative to IB4^-^ t=0 or IB4^+^ t=0. Genes that had relative changes in expression greater than 1.5 fold or less than 0.60 fold were labeled as differentially expressed. The spot intensities were also confirmed visually. Statistical differences between normalized spot intensities of the two neuron populations at both timepoints, and within populations from t=0 to t=24LN, were determined using ANOVA with Tukey post hoc testing and/or Students t-test.

### Quantitative reverse transcriptase polymerase Chain reaction (qRT-PCR)

Select genes from the microarray analysis were further investigated using quantitative RT-PCR to validate possible differential gene expression in IB4^+^ versus non-selected cell populations. The same experiments described above were repeated (n=3 biological replicates), and complementary DNA was synthesized from each RNA sample (IB4^+^ t=0, IB4^-^ t=0, IB4^+^ t=24LN, IB4^-^ t=24LN). Complementary DNA was prepared using a Superscript III First Strand synthesis system for RT-PCR (Invitrogen). The cDNA was quantified using a NanoDrop 1000 spectrophotometer (ThermoScientific). Pre-designed Assays-On-Demand™ Taqman^®^ rat gene expression assays (Applied Biosystems) were used to amplify the target genes (*AdamTs1* – Rn00577887; *CTSH* – Rn00564052*; Fn1* – Rn00569575; *Icam1* – Rn00564227; *Igtb1* – Rn00566727; *Lamb1* – Rn001473698; *Spp1* – Rn00563571; *Plaur* – Rn00569290; *Plat* – Rn00565767). Primer sequences are not provided, but context sequences and amplicon size for each assay are available at Applied BioSystems (Taqman^®^ search: a http://bioinfo.appliedbiosystems.com). Data were expressed relative to IB4^-^ at t=0 using a standard PCR protocol on a Fast Real-Time PCR platform (Applied Biosystems). Relative quantities of gene expression were determined by normalizing to the threshold cycle (CT) values of each gene to the paired *Gapdh* CT values and expressed them relative to IB4^-^ values at t=0 using the relative quantification Comparative CT method (eg. Relative quantity=2^-((IB4+ Gapdh CT at t=0 – paired IB4+GeneCT at t=0) **– (**IB4**-***Gapdh* CT at t=0 **–** paired IB4**-**^^Gene CT at t=0))^). Three biological replicates for each gene were run in triplicate. Statistical significance was calculated using ANOVA with Tukey post hoc testing and/or Students T-test to compare the relative quantity results from each replicated trial.

### Fluorescent immunocytochemistry (ICC)

Total dissociated DRG neurons were cultured on PL and LN coated chambered slides and incubated at 37°C for 45 min (to allow attachment, nominally described as t=0) or 24 hr in NBM. The neurons were then fixed with 4% paraformaldehyde for 20 min, washed three times with phosphate buffered saline (PBS) and blocked in 10% horse serum and 0.1% Triton-X for 1 hr at RT. The neurons were incubated with primary antibodies (see Table 4 for all antibodies employed in this study) specific for laminin (LN), fibronectin (FN), osteopontin (OPN-encoded by the *Spp1* gene), Integrin β1 (ITGβ1), AdamTs1, cathepsin H (CTSH), Plaur, Plat, Icam and β III tubulin overnight at 4°C in a humidified chamber. The slides were washed three times with PBS with 0.1% Tween and incubated with secondary antibodies (Dylight™ donkey anti-mouse 549 (Jackson Immunoresearch), Dylight donkey anti-rabbit 488 (Jackson Immunoresearch) or Alexa donkey anti-goat 488 (Invitrogen), all diluted 1:250 in PBS) for 1 hr at RT. The slides were then washed three times with PBS and incubated in lectin binding buffer (0.1 mM MnCl_2_, 0.1 mM MnCl_2_, 0.1 mM MnCl_2_) for 30 min at RT. The buffer was aspirated and biotinylated isolectin B4 from *Bandeiraea simplicifolia* was added to each well and incubated for 1 hr at RT. Following washing, the wells were incubated with Dylight™ 649 conjugated –Neutravidin (diluted 1:250 in PBS, Thermo Scientific), washed and coverslipped with Gelvatol. Slides were imaged on an Olympus Fluoview FV1000 confocal laser scanning microscope using the z-stage to acquire 1.0 μm optical slices and OIB software to compile z-stacks. Composite images were prepared using Adobe Photoshop, and any alterations to the digital image (eg. for contrast or brightness) were carried out equivalently on t=0 and t=24LN samples. These experiments were repeated 3 to 5 times for each protein.

**Table 4:** List of antibodies employed in this study

**Antibody**	**Source (cat #)**
AdamTs1	AbioTec, 251576, rabbit polyclonal
β III tubulin	Millipore, MAB1637, mouse monoclonal; Sigma T2200, rabbit polyclonal
Basigin/EMMPRIN	Santa Cruz, sc9757, goat polyclonal
Contactin 1	R&D Systems, MAB1714, mouse monoclonal
CTGF	Santa Cruz, sc14939, goat polyclonal
CTSH	Abnova, PAB8634, goat polyclonal
Fibronectin	Abcam, PAB23751, rabbit polyclonal
Icam1(CD54)	Acris, SM286ps, mouse monoclonal
Integrin beta-1	Chemicon, AB1592, rabbit polyclonal
Laminin	Dako, Z0097, rabbit polyclonal; Iowa State Hybridoma Bank 2E8, monoclonal
MMP 19 (MT-MMP)	Santa Cruz, sc12367, goat polyclonal
p75, NGF-R	Millipore, MC192 mouse monoclonal
Plat (tPA)	Santa Cruz, sc5241, goat polyclonal
Plaur (UPAR)	Santa Cruz, sc10815, rabbit polyclonal
SPARC	Santa Cruz, sc25574, rabbit polyclonal
Osteopontin (Spp1)	Abcam, AB8448, rabbit polyclonal
RT1-AW	Santa Cruz, sc71972, mouse monoclonal
Isolectin B4	Sigma, L2140 (biotinylated)

### Densitometric analyses of ICC staining

Confocal images were acquired from slides using equivalent PMT values for each channel. Image stacks were opened in Image J using the Bioformat plugin and the average gray density (0–255 gray levels) and area were manually measured for individual cells. Expression was determined for both IB4+ and IB4- cells and the data then subjected to statistical analyses using 1-way ANOVA (Graph Pad Prism software). The average densities were compared among the various experiments using the tubulin staining signal and the negative control to evaluate slide-slide and experiment-experiment variability. Comparisons were initially assessed between the IB4+ or IB4- cells within a given culture well on a given slide, and then data compared to the different slides/experiments.

### Fluorescent immunohistochemistry (IHC)

DRG were isolated as noted above and immediately frozen in OCT embedding medium in LN_2_. 20 μm sections were obtained and air-dried onto glass slides for subsequent IHC. Sections were fixed in 4% PFA and then subjected to one of the following treatments. Tissue was either blocked with 10% NHS ± 0.1% Triton-X and then processed as outlined above for dissociated cells, or treated with 100% methanol at −20°C for 10 min, washed with PBS and then blocked with 10% NHS and processed as above. Images were acquired with an Olympus Fluoview FV1000 confocal laser scanning microscope using the z-stage to acquire 2.0 μm optical slices and OIB software to compile z-stacks. Composite images were prepared using Adobe Photoshop.

### Data archiving

Array data are deposited in the Dryad repository: http://dx.doi.org/10.5061/dryad.80d7p.

## Abbreviations

Adamts1, AdamTs1: A disintegrin of metalloproteases with thrombospondin-like motifs; Bsg, EMMPRIN: Basigin; Cdh1, Cdh2: Cadherin 1,2; CGRP: Calcitonin gene related peptide; Ctnnd1: Catenin delta 1; Ctsh, CTSH: Cathepsin H; CNS: Central nervous system; Col1a1, Col4a2, Col27a1: Collagen 1a1, 4a2, 27a1; Ctgf: Connective tissue growth factor; Cntn1: Contactin 1; DRG: Dorsal root ganglion; ECM: Extracellular matrix; Fn1, FN: Fibronectin 1; GDNF: Glial derived neurotrophic factor; ICC: Immunocytochemistry; IHC: Immunohistochemistry; Itgb1, ITGB1: Integrin β1; Itgb4: Integrin β4; Icam1: Intercellular adhesion molecule 1; IB4: Isolectin B4; IB4+: IB4 binding neurons; IB4: IB4 negative neurons; LN: Laminin; Lamb1: Laminin β1; Mmp3, Mmp9, Mmp14, Mmp19, Mmp24: Matrix metalloproteinase 3, 9, 14, 19, 24; Ncam1: Neural cell adhesion molecule 1; NF200: Neurofilament 200; OPN: Osteopontin; PNS: Peripheral nervous system; PL: Poly-D-lysine; Spp1: Secreted phosphoprotein 1; Timp1: Tissue inhibitor of metalloproteinases 1; Plat: Tissue plasminogen activator; 2D: Two dimensional; Plaur, Plaur, UPAR: Urokinase plasminogen activator receptor.

## Competing interests

The authors declare that they have no competing interests.

## Authors’ contributions

NF contributed to the majority of the animal surgeries, DRG dissociations, microarray experiments and analyses, real time PCR experiments (analyses and statistics), ICC. KM designed the experiments, performed the ICC analyses and IHC, and revised the final manuscript. Both authors read and approved the final manuscript.

## Supplementary Material

Additional file 1: Table S1 List of ECM genes that were assessed by microarray analysis. Bold type indicates the genes that were detected as being expressed in either or both populations of DRG neurons used in this study. Genes chosen for further analyses were those that were shown to be detected on >3 arrays. Number of biological replicates (and arrays) ranged from 3–7. Click here for file

Additional file 2: Figure S1 Heat maps of gene expression in microarrays. Representative heat maps of 2 different experiments showing differences in gene expression using either the initial arrays (ORN 13) or the second array series (ORN 13.2). Click here for file

Additional file 3: Table S2 36 genes were expressed by both DRG neuron populations. RNA prepared from IB4^+^ and IB4^-^ DRG neurons was analysed using small scale oligonucleotide microarrays. The values presented are the mean spot density from 3–7 different arrays determined as outlined in the Methods; SEM is shown in italics. Eight genes (underlined) were highly expressed in both populations. Nine genes (shown in **bold**) were differentially expressed between the IB4^+^ and IB4^-^ populations at either t=0 or t=24LN. Click here for file

Additional file 4: Table S3 Microarray analysis showed that 21 genes were differentially expressed between the populations or in response to LN. Relative quantity of gene expression was determined by expressing the normalized mean values obtained from microarray analyses relative to the IB4^-^ levels at t=0 or IB4^+^ at t=0. Values that indicated differential expression (defined as >1.5 fold increase or <0.6 fold decrease) are underlined. Nine genes (shown in **bold**) were differentially expressed between the IB4^+^ and IB4^-^ populations at either t=0 or t=24LN, and were chosen for further study. Statistical significance * p<0.05; + p<0.10. Click here for file

Additional file 5: Table S4 qRT-PCR quantitation of gene expression. Nine genes were subjected to qRT-PCR as described in the text and Methods. The data (means + SEM) are expressed relative to the IB4- t=0 condition. Click here for file

Additional file 6: Figure S2 Densitometric analyses of ICC protein expression for selected proteins in dissociated DRG neurons. Quantitation of ICC staining (using average gray level measurements) was performed as outlined in the Methods. Total dissociated neuronal cultures were analysed comparing IB4^+^ vs IB4^-^ cells in the same culture wells, as well as across time points or plating experiments. Statistical significance was noted by ANOVA or Students t-Test. Numbers of cells counted are noted within the bars. Click here for file

Additional file 7: Figure S3 Colour composite images of immunostained DRG sections – series 1. Cryosections of adult rat DRGs were subject to immunohistochemistry for selected proteins (red or green as noted), as well as concomitant labeling with the IB4-lectin (blue). The final column of panels presents the merged images. Scale bar – 50 μm. Click here for file

Additional file 8: Figure S4 Colour composite images of immunostained DRG sections – series 2. Cryosections of adult rat DRGs were subject to immunohistochemistry for selected proteins (red or green as noted), as well as concomitant labeling with the IB4-lectin (blue). The final column of panels presents the merged images. Scale bar – 50 μm. Click here for file
