# Supplementary material for: Extracellular matrix-associated gene expression in adult sensory neuron populations cultured on a laminin substrate
Source: BMC Neurosci. 2013 Jan 30;14:15. doi: 10.1186/1471-2202-14-15 (PMC3610289; doi:10.1186/1471-2202-14-15)
Supplement: Additional file 1: Table S1 — List of ECM genes that were assessed by microarray analysis. Bold type indicates the genes that were detected as being expressed in either or both populations of DRG neurons used in this study. Genes chosen for further analyses were those that were shown to be detected on >3 arrays. Number of biological replicates (and arrays) ranged from 3–7. [file 1471-2202-14-15-S1.doc]

**Supplementary Table 1**

| UniGene | ***RefSeq Number*** | ***Symbol*** | Description |
| --- | --- | --- | --- |
| Rn.7897 | NM_024400 | ***Adamts1*** | ***A disintegrin-like and metallopeptidase (reprolysin type) with***  ***thrombospondin type 1 motif, 1*** |
| Rn.107051 | NM_198761 | Adamts5 | A disintegrin-like and metallopeptidase (reprolysin type) with  thrombospondin type 1 motif, 5 (aggrecanase-2) |
| Rn.100730 | XM_235977 | Adamts8_predicted | A disintegrin-like and metallopeptidase (reprolysin type) with  thrombospondin type 1 motif, 8 (predicted) |
| Rn.2269 | NM_012783 | ***Bsg*** | ***Basigin*** |
| Rn.54474 | NM_022277 | Casp8 | Caspase 8 |
| Rn.32199 | NM_031632 | Casp9 | Caspase 9 |
| Rn.103790 | NM_001007145 | Catna1 | Catenin (cadherin-associated protein), alpha 1 |
| Rn.161845 | XM_232077 | Ctnna2_predicted | Catenin (cadherin associated protein), alpha 2 (predicted) |
| Rn.22518 | NM_031556 | Cav | Caveolin |
| Rn.1120 | NM_012924 | ***Cd44*** | ***CD44 antigen*** |
| Rn.1303 | NM_031334 | ***Cdh1*** | ***Cadherin 1*** |
| Rn.23200 | NM_031333 | ***Cdh2*** | ***Cadherin 2*** |
| Rn.105829 | XM_226426 | Cdh3 | Cadherin 3, type 1, P-cadherin (placental) |
| Rn.161879 | XM_001061943 | Cdh4 | Cadherin 4 |
| Rn.164510 | XM_226213 | Cdh5_predicted | Cadherin 5 (predicted) |
| Rn.91235 | NM_031755 | Ceacam1 | CEA-related cell adhesion molecule 1 |
| Rn.21397 | NM_057118 | ***Cntn1*** | ***Contactin 1*** |
| Rn.260 | XM_342325 | Col11a1 | Procollagen, type XI, alpha 1 |
| Rn.45835 | NM_212528 | Col11a2 | Procollagen, type XI, alpha 2 (mapped) |
| Rn.11218 | XM_243912 | Col12a1 | Procollagen, type XII, alpha 1 |
| Rn.12030 | XM_241632 | Col18a1 | Procollagen, type XVIII, alpha 1 |
| Rn.2953 | XM_213440 | ***Col1a1*** | ***Procollagen, type 1, alpha 1*** |
| Rn.53800 | NM_198747 | ***Col27a1*** | ***Procollagen, type XXVII, alpha 1*** |
| Rn.10124 | NM_012929 | Col2a1 | Procollagen, type II, alpha 1 |
| Rn.3247 | NM_032085 | Col3a1 | Procollagen, type III, alpha 1 |
| Rn.53801 | XM_214400 | ***Col4a1*** | ***Procollagen, type IV, alpha 1*** |
| Rn.2237 | XM_225043 | ***Col4a2_predicted*** | ***Procollagen, type IV, alpha 2 (predicted)*** |
| Rn.121139 | XM_343607 | Col4a3 | Procollagen, type IV, alpha 3 |
| Rn.117 | NM_134452 | Col5a1 | Procollagen, type V, alpha 1 |
| Rn.38654 | NM_021760 | ***Col5a3*** | ***Procollagen, type V, alpha 3*** |
| Rn.107165 | XM_215375 | Col6a1_predicted | Procollagen, type VI, alpha 1 (predicted) |
| Rn.128903 | XM_342115 | Col6a2 | Procollagen, type VI, alpha 2 |
| Rn.24307 | XM_238554 | Col7a1_predicted | Procollagen, type VII, alpha 1 (predicted) |
| Rn.53843 | XM_221536 | Col8a1_predicted | Procollagen, type VIII, alpha 1 (predicted) |
| Rn.90726 | XM_223124 | Col9a1 | Procollagen, type IX, alpha 1 |
| Rn.35666 | XM_215451 | Cspg2 | Chondroitin sulfate proteoglycan 2 |
| Rn.106351 | NM_012837 | ***Cst3*** | ***Cystatin C*** |
| Rn.17145 | NM_022266 | ***Ctgf*** | ***Connective tissue growth factor*** |
| Rn.112601 | NM_053357 | Ctnnb1 | Catenin (cadherin associated protein), beta 1 |
| Rn.9435 | XM_242062 | ***Ctnnd1_predicted*** | ***Catenin (cadherin associated protein), delta 1 (predicted)*** |
| Rn.15163 | XM_001064375 | Ctnnd2 | Catenin (cadherin-associated protein), delta 2 |
| Rn.100909 | NM_022597 | ***Ctsb*** | ***Cathepsin B*** |
| Rn.11085 | NM_134334 | ***Ctsd*** | ***Cathepsin D*** |
| Rn.92738 | NM_012938 | Ctse | Cathepsin E |
| Rn.103332 | XM_214205 | Ctsg_predicted | Cathepsin G (predicted) |
| Rn.1997 | NM_012939 | ***Ctsh*** | ***Cathepsin H*** |
| Rn.1294 | NM_013156 | ***Ctsl*** | ***Cathepsin L*** |
| Rn.10666 | NM_012841 | Dcc | Deleted in colorectal carcinoma |
| Rn.97792 | NM_053882 | Ecm1 | Extracellular matrix protein 1 |
| Rn.99346 | XM_238447 | Emilin1_predicted | Elastin microfibril interfacer 1 (predicted) |
| Rn.17491 | NM_022587 | Entpd1 | Ectonucleoside triphosphate diphosphohydrolase 1 |
| Rn.9375 | XM_243637 | Fbln1_predicted | Fibulin 1 (predicted) |
| Rn.11416 | NM_020071 | Fgb | Fibrinogen, B beta polypeptide |
| Rn.1604 | NM_019143 | ***Fn1*** | ***Fibronectin 1*** |
| Rn.107273 | NM_053796 | F11r | F11 receptor |
| Rn.50531 | NM_019189 | Hapln1 | Hyaluronan and proteoglycan link protein 1 |
| Rn.52931 | XM_222716 | Hmcn1_predicted | Hemicentin 1 (predicted) |
| Rn.6392 | NM_022605 | ***Hpse*** | ***Heparanase*** |
| Rn.12 | NM_012967 | ***Icam1*** | ***Intercellular adhesion molecule 1*** |
| Rn.91044 | NM_030994 | Itga1 | Integrin alpha 1 |
| Rn.82866 | XM_227469 | Itga10_predicted | Integrin, alpha 10 (predicted) |
| Rn.64478 | XM_236320 | Itga11_predicted | Integrin, alpha 11 (predicted) |
| Rn.83597 | XM_345156 | Itga2 | Integrin, alpha 2 |
| Rn.154664 | XM_340884 | Itga3_predicted | Integrin alpha 3 (predicted) |
| Rn.12704 | XM_230033 | Itga4 | Integrin alpha 4 (mapped) |
| Rn.100796 | XM_235707 | ***Itga5*** | ***Integrin alpha 5 (mapped)*** |
| Rn.161799 | XM_215984 | Itga6 | Integrin, alpha 6 |
| Rn.54492 | NM_030842 | Itga7 | Integrin alpha 7 |
| Rn.69726 | XM_225600 | Itga8 | Integrin alpha 8 |
| Rn.34728 | NM_031691 | Itgad | Integrin, alpha D |
| Rn.29975 | NM_031768 | Itgae | Integrin, alpha E, epithelial-associated |
| Rn.14655 | NM_001033998 | Itgal | Integrin alpha L |
| Rn.54465 | NM_012711 | Itgam | Integrin alpha M |
| Rn.23339 | XM_230950 | Itgav_predicted | Integrin alpha V (predicted) |
| Rn.25733 | NM_017022 | ***Itgb1*** | ***Integrin beta 1 (fibronectin receptor beta)*** |
| Rn.42962 | XM_001069791 | Itgb2 | Integrin beta 2 |
| Rn.162202 | NM_153720 | Itgb3 | Integrin beta 3 |
| Rn.198908 | NM_013180 | ***Itgb4*** | ***Integrin beta 4*** |
| Rn.16988 | NM_147139 | Itgb5 | Integrin, beta 5 |
| Rn.19828 | NM_001004263 | Itgb6 | Integrin, beta 6 |
| Rn.10767 | XM_343336 | Itgb7 | Integrin, beta 7 |
| Rn.72676 | XM_343126 | Itgb8_predicted | Integrin beta 8 (predicted) |
| Rn.2807 | XM_237536 | Lama1_predicted | Laminin, alpha 1 (predicted) |
| Rn.21475 | XM_219866 | Lama2_predicted | Laminin, alpha 2 (predicted) |
| Rn.10597 | XM_226159 | Lama3 | Laminin, alpha 3 |
| Rn.99620 | XM_228366 | Lama4_predicted | Laminin, alpha 4 (predicted) |
| Rn.62616 | XM_215963 | Lama5 | Laminin, alpha 5 |
| Rn.48387 | XM_216679 | ***Lamb1_predicted*** | ***Laminin, beta 1 (predicted)*** |
| Rn.774 | NM_012974 | Lamb2 | Laminin, beta 2 |
| Rn.49634 | XM_223087 | Lamb3 | Laminin, beta 3 |
| Rn.7145 | XM_341133 | Lamc1 | Laminin, gamma 1 |
| Rn.20927 | XM_001062937 | LOC685233 | Hypothetical protein LOC685233 |
| Rn.198228 | XM_001061798 | LOC501069 | Similar to golgi autoantigen golgin subtype a4; tGolgin-1 |
| Rn.162539 | NM_131904 | Mgea5 | Meningioma expressed antigen 5 (hyaluronidase) |
| Rn.9946 | NM_133514 | Mmp10 | Matrix metallopeptidase 10 |
| Rn.11123 | NM_012980 | Mmp11 | Matrix metallopeptidase 11 |
| Rn.33193 | NM_053963 | Mmp12 | Matrix metallopeptidase 12 |
| Rn.10997 | XM_343345 | ***Mmp13*** | ***Matrix metallopeptidase 13*** |
| Rn.10371 | NM_031056 | ***Mmp14*** | ***Matrix metallopeptidase 14 (membrane-inserted)*** |
| Rn.165433 | XM_238034 | Mmp15_predicted | Matrix metallopeptidase 15 (predicted) |
| Rn.118859 | NM_080776 | Mmp16 | Matrix metalloproteinase 16 |
| Rn.103646 | XM_239639 | Mmp17_predicted | Matrix metallopeptidase 17 (predicted) |
| Rn.21771 | XM_222317 | ***Mmp19_predicted*** | ***Matrix metalloproteinase 19 (predicted)*** |
| Rn.79007 | XM_235794 | Mmp1a_predicted | Matrix metallopeptidase 1a (interstitial collagenase) (predicted) |
| Rn.6422 | NM_031054 | Mmp2 | Matrix metallopeptidase 2 |
| N/A | XM_235796 | Mmp20_predicted | Matrix metalloproteinase 20 (enamelysin) (predicted) |
| Rn.22562 | NM_053606 | Mmp23 | Matrix metallopeptidase 23 |
| Rn.3117 | NM_031757 | ***Mmp24*** | ***Matrix metallopeptidase 24*** |
| Rn.32086 | NM_133523 | Mmp3 | Matrix metallopeptidase 3 |
| Rn.10282 | NM_012864 | Mmp7 | Matrix metallopeptidase 7 |
| Rn.44474 | NM_022221 | Mmp8 | Matrix metallopeptidase 8 |
| Rn.10209 | NM_031055 | Mmp9 | Matrix metallopeptidase 9 |
| Rn.64144 | XM_342281 | Muc1 | Mucin 1, transmembrane |
| Rn.11283 | NM_031521 | ***Ncam1*** | ***Neural cell adhesion molecule 1*** |
| Rn.138756 | NM_203409 | Ncam2 | Neural cell adhesion molecule 2 |
| Rn.10691 | NM_013150 | Nrcam | Neuron-glia-CAM-related cell adhesion molecule |
| Rn.1878 | NM_031591 | Pecam | Platelet/endothelial cell adhesion molecule |
| Rn.107102 | NM_013151 | ***Plat*** | ***Plasminogen activator, tissue*** |
| Rn.82711 | NM_017350 | ***Plaur*** | ***Plasminogen activator, urokinase receptor*** |
| Rn.6064 | NM_013085 | Plau | Plasminogen activator, urokinase |
| Rn.30516 | XM_342245 | Postn_predicted | Periostin, osteoblast specific factor (predicted) |
| Rn.116787 | NM_012645 | ***RT1-Aw2*** | ***RT1 class Ib, locus Aw2*** |
| Rn.10359 | NM_138879 | Sele | Selectin, endothelial cell |
| Rn.10461 | NM_019177 | Sell | Selectin, lymphocyte |
| Rn.10012 | NM_013114 | Selp | Selectin, platelet |
| Rn.25752 | NM_057108 | Serpinb5 | Serine (or cysteine) peptidase inhibitor, clade B, member 5 |
| Rn.29367 | NM_012620 | Serpine1 | Serine (or cysteine) peptidase inhibitor, clade E, member 1 |
| Rn.2271 | XM_343604 | Serpine2 | Serine (or cysteine) proteinase inhibitor, clade E, member 2 |
| Rn.185815 | NM_001002023 | Sgce | Sarcoglycan, epsilon |
| Rn.98989 | NM_012656 | ***Sparc*** | ***Secreted acidic cysteine rich glycoprotein*** |
| Rn.44057 | XM_225160 | Spock1 | Sparc/osteonectin, cwcv and kazal-like domains proteoglycan 1 |
| Rn.8871 | NM_012881 | ***Spp1*** | ***Secreted phosphoprotein 1*** |
| Rn.7596 | NM_001033680 | Syt1 | Synaptotagmin I |
| Rn.1046 | XM_573983 | Tgfbi | Transforming growth factor, beta induced |
| Rn.185771 | NM_001013062 | Thbs1 | Thrombospondin 1 |
| Rn.165619 | XM_214778 | Thbs2 | Thrombospondin 2 |
| Rn.11207 | XM_342172 | Thbs4 | Thrombospondin 4 |
| Rn.25754 | NM_053819 | ***Timp1*** | ***Tissue inhibitor of metallopeptidase 1*** |
| Rn.10161 | NM_021989 | Timp2 | Tissue inhibitor of metalloproteinase 2 |
| Rn.119634 | NM_012886 | Timp3 | Tissue inhibitor of metalloproteinase 3 (Sorsby fundus dystrophy, pseudoinflammatory) |
| N/A | XM_345932 | Tmprss4_predicted | Transmembrane protease, serine 4 (predicted) |
| Rn.12723 | U15550 | Tnc | Tenascin C |
| Rn.11267 | NM_012889 | Vcam1 | Vascular cell adhesion molecule 1 |
| Rn.87493 | NM_019156 | Vtn | Vitronectin |
