# Supplementary figures and images for: Extracellular matrix-associated gene expression in adult sensory neuron populations cultured on a laminin substrate
Source: BMC Neurosci. 2013 Jan 30;14:15. doi: 10.1186/1471-2202-14-15 (PMC3610289; doi:10.1186/1471-2202-14-15)

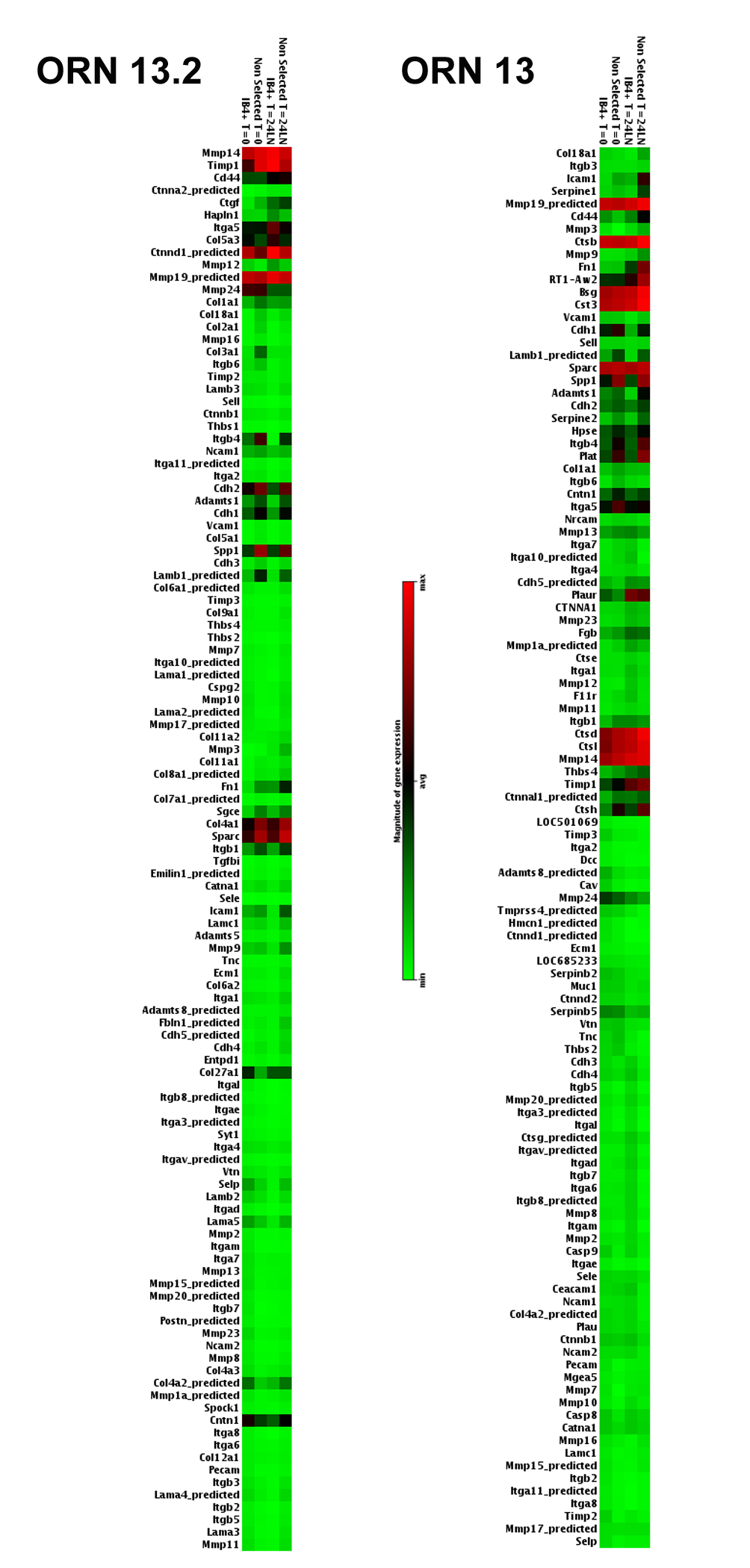

Supplement: Additional file 2: Figure S1 — Heat maps of gene expression in microarrays. Representative heat maps of 2 different experiments showing differences in gene expression using either the initial arrays (ORN 13) or the second array series (ORN 13.2). [file 1471-2202-14-15-S2.tiff]

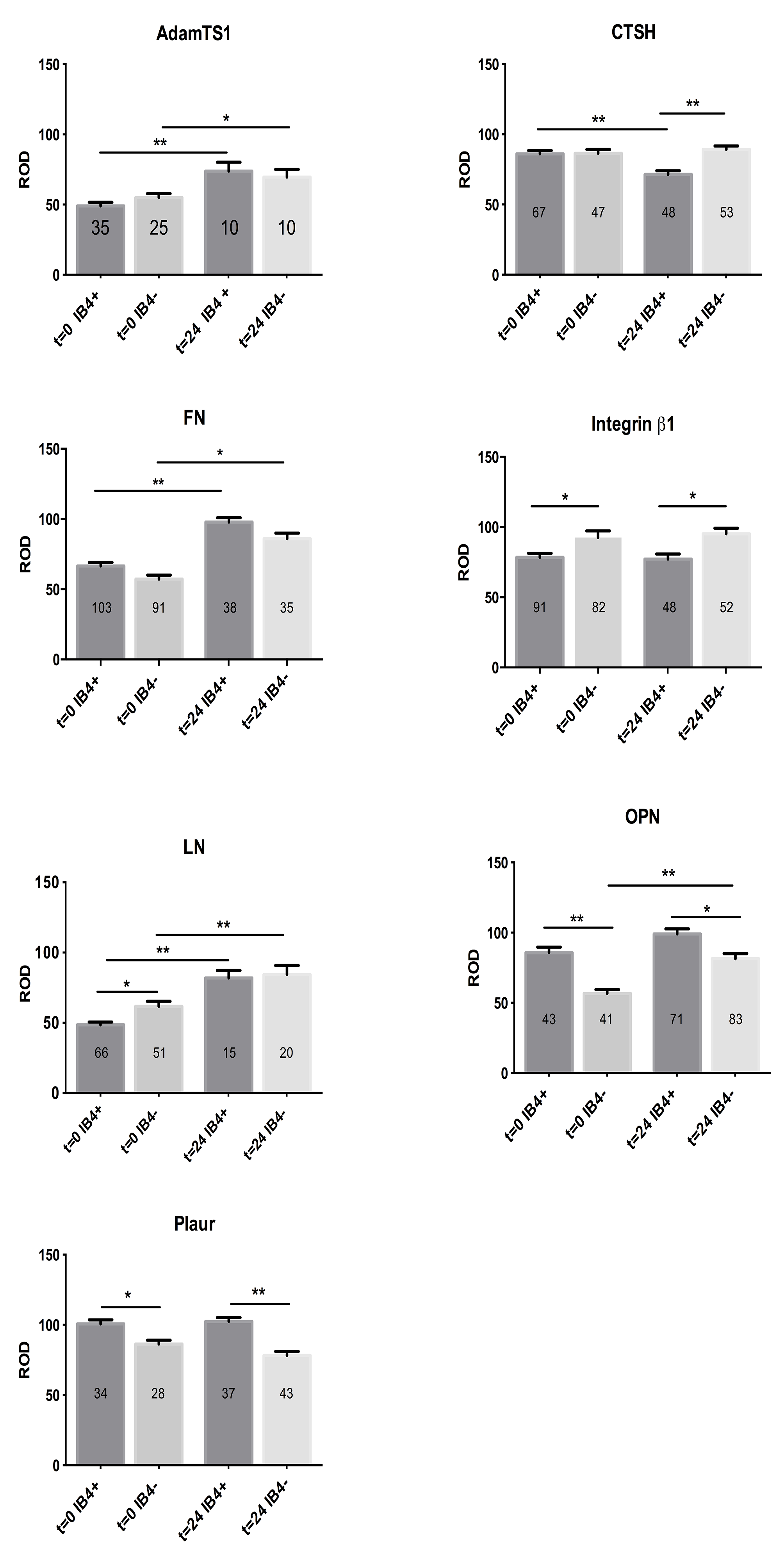

Supplement: Additional file 6: Figure S2 — Densitometric analyses of ICC protein expression for selected proteins in dissociated DRG neurons. Quantitation of ICC staining (using average gray level measurements) was performed as outlined in the Methods. Total dissociated neuronal cultures were analysed comparing IB4+ vs IB4- cells in the same culture wells, as well as across time points or plating experiments. Statistical significance was noted by ANOVA or Students t-Test. Numbers of cells counted are noted within the bars. [file 1471-2202-14-15-S6.tiff]

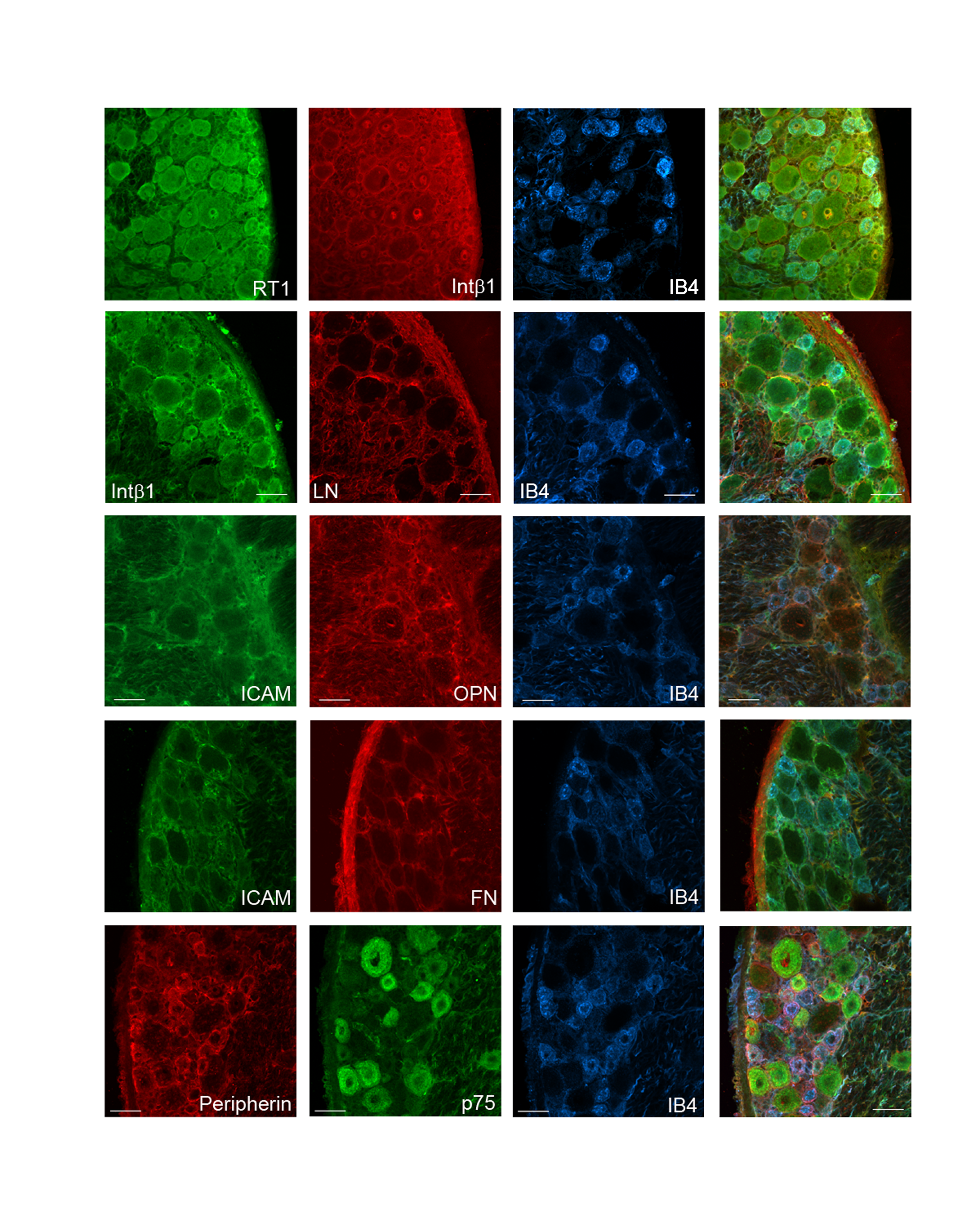

Supplement: Additional file 7: Figure S3 — Colour composite images of immunostained DRG sections – series 1. Cryosections of adult rat DRGs were subject to immunohistochemistry for selected proteins (red or green as noted), as well as concomitant labeling with the IB4-lectin (blue). The final column of panels presents the merged images. Scale bar – 50 μm. [file 1471-2202-14-15-S7.tiff]

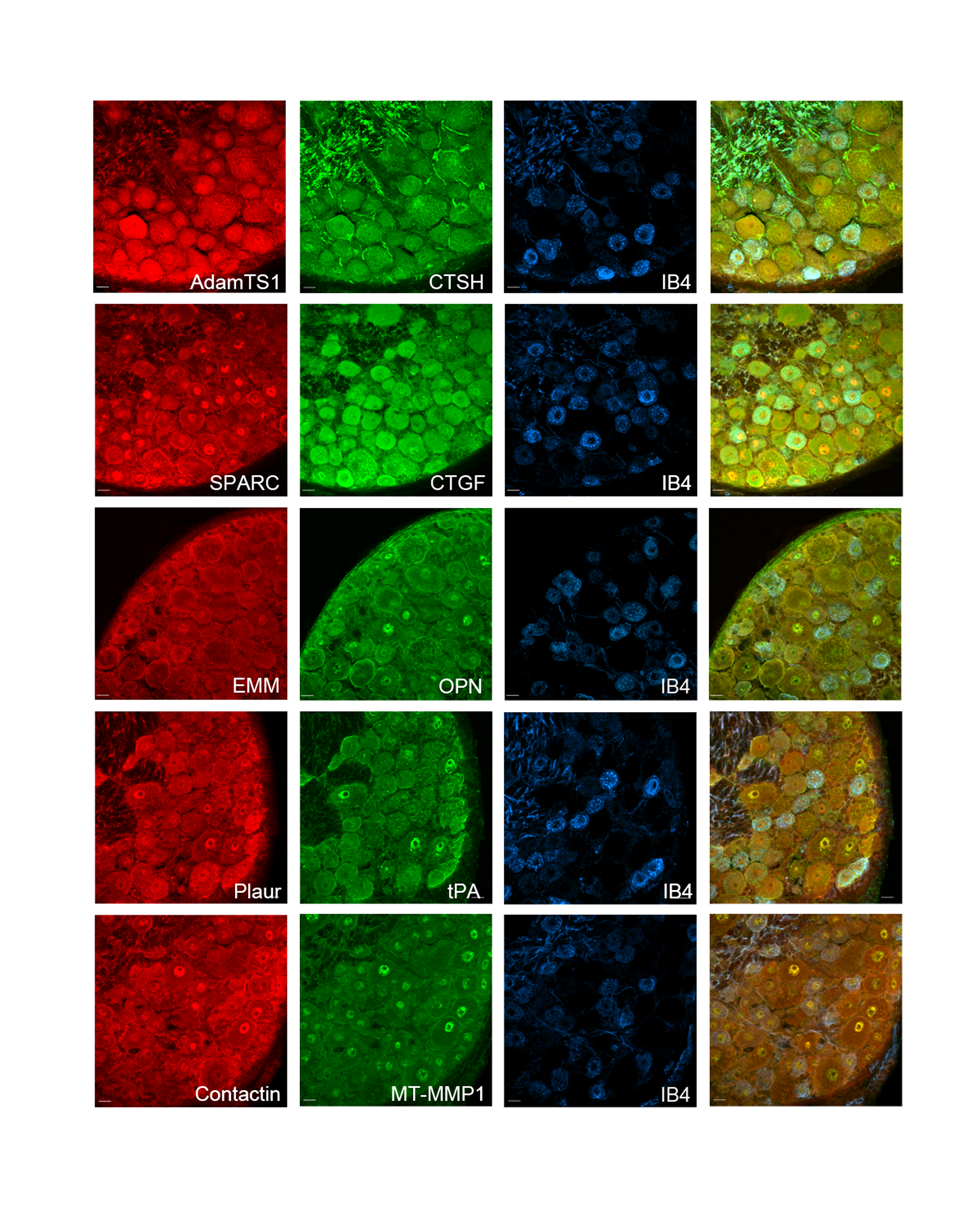

Supplement: Additional file 8: Figure S4 — Colour composite images of immunostained DRG sections – series 2. Cryosections of adult rat DRGs were subject to immunohistochemistry for selected proteins (red or green as noted), as well as concomitant labeling with the IB4-lectin (blue). The final column of panels presents the merged images. Scale bar – 50 μm. [file 1471-2202-14-15-S8.tiff]
