# Supplementary material for: Extracellular matrix-associated gene expression in adult sensory neuron populations cultured on a laminin substrate
Source: BMC Neurosci. 2013 Jan 30;14:15. doi: 10.1186/1471-2202-14-15 (PMC3610289; doi:10.1186/1471-2202-14-15)
Supplement: Additional file 3: Table S2 — 36 genes were expressed by both DRG neuron populations. RNA prepared from IB4+ and IB4- DRG neurons was analysed using small scale oligonucleotide microarrays. The values presented are the mean spot density from 3–7 different arrays determined as outlined in the Methods; SEM is shown in italics. Eight genes (underlined) were highly expressed in both populations. Nine genes (shown in bold) were differentially expressed between the IB4+ and IB4- populations at either t=0 or t=24LN. [file 1471-2202-14-15-S3.doc]

Supplementary Table 2. 36 genes were expressed by both DRG neuron populations. The values shown are the mean (with *SEM*) of 3-7 array replicates (spot densities normalized to the housekeeping genes on the arrays). 21 of these were differentially expressed between the groups (***bold***). Genes underlined were expressed at relatively high (compared to housekeeping genes) levels but did not differ between the IB4+ vs IB4- cells.

| **Receptor/Adhesion molecules** | | | | | |
| --- | --- | --- | --- | --- | --- |
| **Gene symbol** | **Gene Name** | **IB4^+^**  **t=0** | **IB4^-^**  **t=0** | **IB4^+^**  **t=24LN** | **IB4^-^**  **t=24LN** |
| ***Icam1*** | **Intercellular adhesion molecule** | 0.11  *(0.04)* | 0.21  *(0.07)* | 0.11  *(0.05)* | 0.49  *(0.15)* |
| *Itga5* | Integrin alpha 5 | 0.63  *(0.13)* | 0.88  *(0.11)* | 0.67  *(0.14)* | 0.71  *(0.15)* |
| ***Itgb1*** | **Integrin beta 1** | 0.30  *(0.08)* | 0.71  *(0.14)* | 0.24  *(0.07)* | 0.36  *(0.06)* |
| *Itgb4* | Integrin beta 4 | 0.50  *(0.09)* | 0.78  *(0.05)* | 0.33  *(0.06)* | 0.67  *(0.12)* |
| *Cdh1* | Cadherin 1 | 0.47  *(0.07)* | 0.63  *(0.09)* | 0.21  *(0.03)* | 0.53  *(0.14)* |
| *Cdh2* | Cadherin 2 | 0.38  *(0.04)* | 0.57  *(0.08)* | 0.35  *(0.08)* | 0.65  *(0.14)* |
| *Ctnnd1_predicted* | Catenin, delta 1 predicted | 0.4  *(0.19)* | 0.42  *(0.18)* | 0.61  *(0.04)* | 0.73  *(0.14)* |
| *Cntn1* | Contactin 1 | 0.50  *(0.08)* | 0.51  *(0.05)* | 0.36  *(0.07)* | 0.60  *(0.11)* |
| *Cd44* | CD44 antigen | 0.34  *(0.06)* | 0.20  *(0.06)* | 0.44  *(0.09)* | 0.59  *(0.14)* |
| ***Plaur*** | **Plasminogen activator, urokinase receptor** | 0.41  *(0.05)* | 0.28  *(0.02)* | 0.98  *(0.12)* | 0.57  *(0.19)* |
| *Ncam1* | Neural cell adhesion molecule 1 | 0.06  *(0.02)* | 0.15  *(0.05)* | 0.11  *(0.04)* | 0.12  *(0.04)* |
| *RT1-Aw2* | RT1 class Ib, locus Aw2 | 0.53  *(0.16)* | 0.55  *(0.11)* | 0.75  *(0.02)* | 1.01  *(0.07)* |

| **ECM proteins** | | | | | |
| --- | --- | --- | --- | --- | --- |
| **Gene symbol** | **Gene Name** | **IB4^+^**  **t=0** | **IB4^-^**  **t=0** | **IB4^+^**  **t=24LN** | **IB4^-^**  **t=24LN** |
| ***Fn1*** | **Fibronectin 1** | 0.1  *(0.04)* | 0.19  *(0.07)* | 0.33  *(0.10)* | 0.73  *(0.19)* |
| ***Spp1*** | **Secreted phosphoprotein 1 (osteonectin)** | 0.52  *(0.14)* | 0.90  *(0.09)* | 0.48  *(0.06)* | 0.82  *(0.10)* |
| ***Lamb1_predicted*** | **Laminin, beta 1 predicted** | 0.18  *(0.05)* | 0.46  *(0.09)* | 0.08  *(0.01)* | 0.36  *(0.13)* |
| *Col1a1* | Procollagen, type 1, α1 | 0.16  *(0.05)* | 0.29  *(0.08)* | 0.19  (0.06) | 0.22  *(0.06)* |
| *Col4a1* | Procollagen, type 4, α1 | 0.42  *(0.12)* | 0.70  *(0.11)* | 0.62  *(0.21)* | 0.81  *(0.09)* |
| *Col4a2_predicted* | Procollagen, type 4, α2  predicted | 0.19  *(0.09)* | 0.12  *(0.04)* | 0.13  *(0.06)* | 0.24  *(0.11)* |
| *Col5a3* | Procollagen, type 5, α3 | 0.65  *(0.17)* | 0.41  *(0.11)* | 0.6  *(0.21)* | 0.51  *(0.20)* |
| *Col27a1* | Procollagen, type XXVII, α1 | 0.57  *(0.25)* | 0.17  *(0.09)* | 0.35  *(0.20)* | 0.44  *(0.21)* |

| **Lysosomal proteases** | | | | | |
| --- | --- | --- | --- | --- | --- |
| **Gene symbol** | **Gene Name** | **IB4^+^**  **t=0** | **IB4^-^**  **t=0** | **IB4^+^**  **t=24LN** | **IB4^-^**  **t=24LN** |
| Ctsb | Cathepsin B | 1.21  *(0.09)* | 1.18  *(0.03)* | 1.22  *(0.02)* | 1.34  *(0.12)* |
| Ctsd | Cathepsin D | 1.03  *(0.08)* | 1.13  *(0.05)* | 1.19  *(0.03)* | 1.31  *(0.11)* |
| ***Ctsh*** | **Cathepsin H** | 0.33  *(0.07)* | 0.71  *(0.03)* | 0.46  *(0.05)* | 0.88  *(0.06)* |
| *Ctsl* | Cathepsin L | 1.01  *(0.15)* | 1.14  *(0.03)* | 1.18  *(0.05)* | 1.27  *(0.07)* |

| **Matrix Metallopeptidases (MMPs)** | | | | | |
| --- | --- | --- | --- | --- | --- |
| **Gene symbol** | **Gene Name** | **IB4^+^**  **t=0** | **IB4^-^**  **t=0** | **IB4^+^**  **t=24LN** | **IB4^-^**  **t=24LN** |
| *Mmp13* | Matrix Metallopeptidase 13 | 0.23  *(0.06)* | 0.3  *(0.08)* | 0.32  *(0.09)* | 0.26  *(0.05)* |
| *Mmp14* | Matrix Metallopeptidase 14 | 1.01  *(0.07)* | 1.05  *(0.05)* | 1.1  *(0.06)* | 1.03  *(0.11)* |
| *Mmp19* | Matrix Metallopeptidase 19 | 1.07  *(0.08)* | 0.99  *(0.10)* | 1.08  *(0.07)* | 1.04  *(0.11)* |
| *Mmp24* | Matrix metallopeptidase 24 | 0.57  *(0.09)* | 0.54  *(0.09)* | 0.33  (0.08) | 0.31  *(0.06)* |

| Regulators of MMPs | | | | | |
| --- | --- | --- | --- | --- | --- |
| **Gene symbol** | **Gene Name** | **IB4^+^**  **t=0** | **IB4^-^**  **t=0** | **IB4^+^**  **t=24LN** | **IB4^-^**  **t=24LN** |
| *Bsg* | Basigin | 1.11  *(0.08)* | 1.16  *(0.02)* | 1.19  *(0.02)* | 1.32  *(0.11)* |
| *Cst3* | Cystatin 3 | 1.15  *(0.12)* | 1.18  *(0.03)* | 1.22  *(0.02)* | 1.34  *(0.12)* |
| *Ctgf* | Connective tissue growth factor | 0.04  *(0.02)* | 0.17  *(0.09)* | 0.3  *(0.13)* | 0.39  *(0.20)* |
| *Sparc* | Secreted acidic cysteine rich glycoprotein | 0.91  *(0.14)* | 0.97  *(0.11)* | 0.84  *(0.14)* | 0.98  *(0.07)* |
| *Timp1* | Tissue inhibitor of metallopeptidase 1 | 0.54  *(0.09)* | 0.82  *(0.10)* | 0.97  *(0.03)* | 0.91  *(0.11)* |

| **Other extracellular proteases** | | | | | |
| --- | --- | --- | --- | --- | --- |
| **Gene symbol** | **Gene Name** | **IB4^+^**  **t=0** | **IB4^-^**  **t=0** | **IB4^+^**  **t=24LN** | **IB4^-^**  **t=24LN** |
| *Adamts1* | **A disintegrin-like and metallopeptidase (reprolysin type) with thrombospondin type 1 motif, 1** | 0.26  *(0.07)* | 0.37  *(0.12)* | 0.10  *(0.03)* | 0.50  *(0.15)* |
| *Hpse* | Heparanase | 0.43  *(0.12)* | 0.57  *(0.06)* | 0.45  *(0.04)* | 0.66  *(0.13)* |
| ***Plat*** | **Plasminogen activator, tissue** | 0.44  *(0.07)* | 0.78  *(0.04)* | 0.58  *(0.15)* | 1.01  *(0.01)* |
