# Supplementary material for: Extracellular matrix-associated gene expression in adult sensory neuron populations cultured on a laminin substrate
Source: BMC Neurosci. 2013 Jan 30;14:15. doi: 10.1186/1471-2202-14-15 (PMC3610289; doi:10.1186/1471-2202-14-15)
Supplement: Additional file 4: Table S3 — Microarray analysis showed that 21 genes were differentially expressed between the populations or in response to LN. Relative quantity of gene expression was determined by expressing the normalized mean values obtained from microarray analyses relative to the IB4- levels at t=0 or IB4+ at t=0. Values that indicated differential expression (defined as >1.5 fold increase or <0.6 fold decrease) are underlined. Nine genes (shown in bold) were differentially expressed between the IB4+ and IB4- populations at either t=0 or t=24LN, and were chosen for further study. Statistical significance * p<0.05; + p<0.10. [file 1471-2202-14-15-S4.doc]

**Supplementary Table 3:** Microarray analysis showed that 21 genes were differentially expressed between IB4^+^ and IB4^-^ DRG neurons. Values are the relative expression of IB4+ to IB4- at t=0 or t=24, or IB4+ 24/0 and IB4-24/0. Differential expression was defined as > 1.5 fold increase or < 0.6 fold decrease (underlined values). Differences that are statistically significant are noted by * (p<0.05) or + (p<0.1).

| Gene symbol | Gene Name | IB4^+^ t=0/ IB4^-^ t=0 | IB4^+^ t=24LN/ IB4^+^ t=0 | IB4^-^ t=24LN/ IB4^-^ t=0 | IB4^+^ t=24LN/ IB4^-^ t=24LN |
| --- | --- | --- | --- | --- | --- |
| *Adamts1* | **A disintegrin-like and metallopeptidase (reprolysin type) with thrombospondin type 1 motif, 1** | 0.70 | 0.38 | 1.35 | 0.20^+^ |
| *Cd44* | CD44 antigen | 1.55 | 1.42 | 2.95* | 0.75 |
| *Cdh1* | Cadherin 1 | 0.75 | 0.47 | 0.70 | 0.4 |
| *Cdh2* | Cadherin 2 | 0.67 | 0.92 | 1.14 | 0.54 |
| *Col1a1* | Procollagen, type 1, α1 | 0.55 | 1.19 | 0.76 | 0.86 |
| *Col27a1* | Procollagen, type XXVII, α1 | 3.35 | 0.61 | 2.59 | 0.80 |
| *Col4a2*  *predicted* | Procollagen, type 4, α2 predicted | 1.58 | 0.68 | 2 | 0.54 |
| *Cntn1* | Contactin 1 | 0.98 | 0.72 | 1.18 | 0.60 |
| *Ctgf* | Connective tissue growth factor | 0.23 | 7.5 | 2.29 | 0.77 |
| ***Ctsh*** | **Cathepsin H** | 0.46* | 1.39 | 1.23 | 0.52* |
| ***Fn1*** | **Fibronectin 1** | 0.52 | 3.30 | 3.80^+^ | 0.45 |
| Icam1 | **Intercellular adhesion molecule 1** | 0.52 | 1.0 | 2.33 | 0.22* |
| ***Itgb1*** | **Integrin beta 1** | 0.42* | 0.8 | 0.51^+^ | 0.67 |
| *Itgb4* | Integrin beta 4 | 0.64 | 0.66 | 0.85 | 0.49* |
| ***Lamb1***  ***Pred.*** | **Laminin, beta 1 predicted** | 0.39^+^ | 0.44 | 0.78 | 0.22^+^ |
| *Ncam1* | Neural cell adhesion molecule 1 | 0.4 | 1.83 | 0.8 | 0.92 |
| ***Plat*** | **Plasminogen activator, tissue** | 0.57* | 1.23 | 1.23 | 0.57* |
| Plaur | **Plasminogen activator, urokinase receptor** | 1.46 | 2.40* | 2.03 | 1.72^+^ |
| ***Spp1*** | **Secreted phosphoprotein 1 (osteopontin)** | 0.58^+^ | 0.92 | 0.91 | 0.58^+^ |
| *RT-AW2* | RT1 class 1b, locus Aw2 | 0.96 | 1.41 | 1.65* | 0.82 |
| *Timp1* | Tissue inhibitor of metallopeptidase 1 | 0.65 | 1.79* | 1.10 | 1.06 |
