# Supplementary material for: Extracellular matrix-associated gene expression in adult sensory neuron populations cultured on a laminin substrate
Source: BMC Neurosci. 2013 Jan 30;14:15. doi: 10.1186/1471-2202-14-15 (PMC3610289; doi:10.1186/1471-2202-14-15)
Supplement: Additional file 5: Table S4 — qRT-PCR quantitation of gene expression. Nine genes were subjected to qRT-PCR as described in the text and Methods. The data (means + SEM) are expressed relative to the IB4- t=0 condition. [file 1471-2202-14-15-S5.doc]

Supplementary Table 4. Relative gene expression as determined

from qRT-PCR analyses (expressed relative to IB4- t=0).

| **Genes** | IB4+ t=0 | IB+ t=24 LN | IB4- t=24 LN |
| --- | --- | --- | --- |
| *Adamts1* | 0.44*  *(0.06)* | 0.28*  *(0.14)* | 0.15*  *(0.03)* |
| *Ctsh* | 0.53  *(0.29)* | 0.68  *(0.35)* | 0.19^+^  *(0.07)* |
| *Fn1* | 0.35  *(0.03)* | 1.67  *(0.80)* | 0.87  *(0.27)* |
| *Icam1* | 0.44  *(0.09)* | 0.52  *(0.22)* | 0.38^+^  *(0.09)* |
| *Itgb1* | 1.14  *(0.05)* | 1.85^+^  *(0.30)* | 1.63  *(0.14)* |
| *Lamb1* | 0.38*  *(0.07)* | 0.32  *(0.12)* | 0.36*  *(0.08)* |
| *Plat* | 0.45^+^  *(0.06)* | 1.14  *(0.42)* | 0.94  *(0.24)* |
| *Plaur* | 2.35  *(0.17)* | 8.02*  *(2.24)* | 5.55^+^  *(0.60)* |
| *Spp1* | 0.61  *(0.32)* | 0.87  *(0.40)* | 0.48  *(0.14)* |

Values are Mean + *(SEM).* Statistical significance was determined

using one-way ANOVA with p <0.05(*) or p <0.1(+).
